# Supplementary material for: Fungal kinases and transcription factors regulating brain infection in Cryptococcus neoformans
Source: Nat Commun. 2020 Mar 23;11:1521. doi: 10.1038/s41467-020-15329-2 (PMC7090016; doi:10.1038/s41467-020-15329-2)
Supplement: Supplementary file 1 — Supplementary Information [file 41467_2020_15329_MOESM1_ESM.pdf]

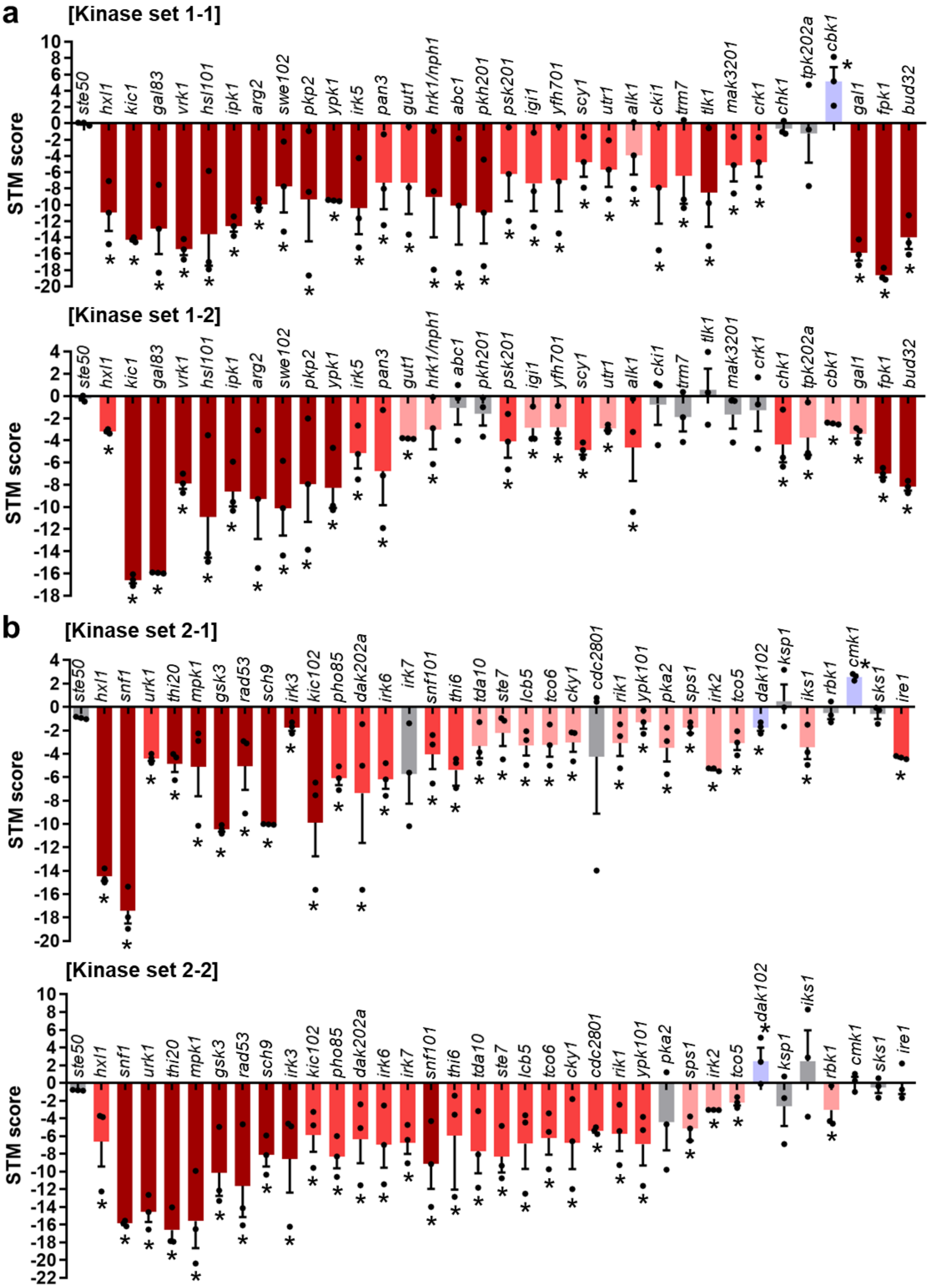
a
[Kinase set 1-1]
STM score
ste50 hxl1 kic1 gal83 vrk1 hsl101 ipk1 arg2 swe102 pkp2 ypk1 irk5 pan3 gut1 hrk1/nph1 abc1 pkh201 psk201 igi1 yfh701 scy1 utr1 alk1 cki1 trm7 tlk1 mak3201 crk1 chk1 tpk202a cbk1 gal1 fpk1 bud32
[Kinase set 1-2]
STM score
ste50 hxl1 kic1 gal83 vrk1 hsl101 ipk1 arg2 swe102 pkp2 ypk1 irk5 pan3 gut1 hrk1/nph1 abc1 pkh201 psk201 igi1 yfh701 scy1 utr1 alk1 cki1 trm7 tlk1 mak3201 crk1 chk1 tpk202a cbk1 gal1 fpk1 bud32
b
[Kinase set 2-1]
STM score
ste50 hxl1 snf1 urk1 thi20 mpk1 gsk3 rad53 sch9 irk3 kic102 pho85 dak202a irk6 irk7 snf101 thi6 tda10 ste7 lcb5 tco6 cky1 cdc2801 rik1 ypk101 pka2 sps1 irk2 tco5 dak102 ksp1 iks1 rbk1 cmk1 sks1 ire1
[Kinase set 2-2]
STM score
ste50 hxl1 snf1 urk1 thi20 mpk1 gsk3 rad53 sch9 irk3 kic102 pho85 dak202a irk6 irk7 snf101 thi6 tda10 ste7 lcb5 tco6 cky1 cdc2801 rik1 ypk101 pka2 sps1 irk2 tco5 dak102 ksp1 iks1 rbk1 cmk1 sks1 ire1

Continued

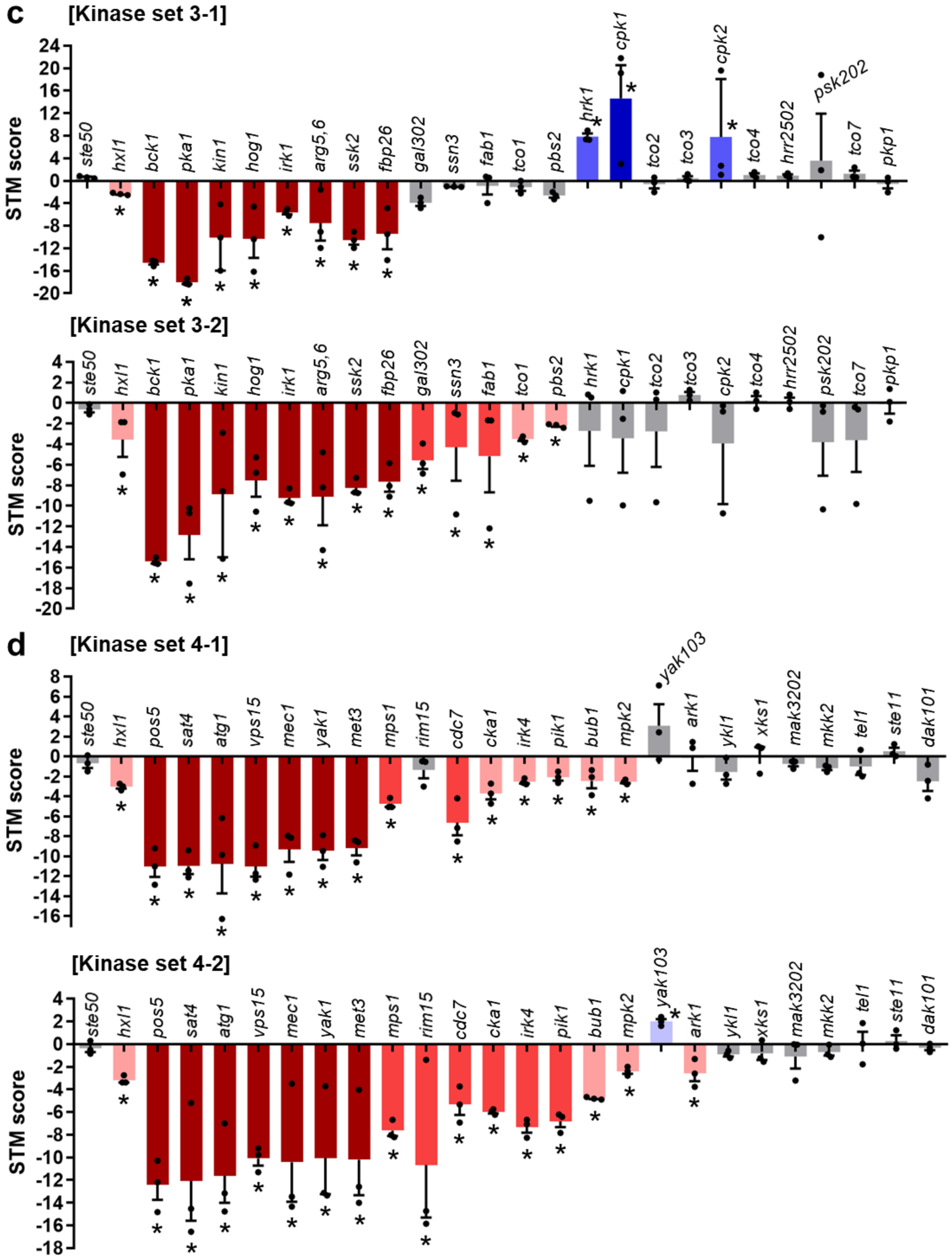
c
[Kinase set 3-1]
STM score
ste50 hxl1 bck1 pka1 kin1 hog1 irk1 arg5,6 ssk2 fbp26 gal302 ssn3 fab1 tco1 pbs2 hrk1 cpk1 tco2 tco3 cpk2 tco4 hrr2502 psk202 tco7 pkp1
[Kinase set 3-2]
STM score
ste50 hxl1 bck1 pka1 kin1 hog1 irk1 arg5,6 ssk2 fbp26 gal302 ssn3 fab1 tco1 pbs2 hrk1 cpk1 tco2 tco3 cpk2 tco4 hrr2502 psk202 tco7 pkp1
d
[Kinase set 4-1]
STM score
ste50 hxl1 pos5 sat4 atg1 vps15 mec1 yak1 met3 mps1 rim15 cdc7 cka1 irk4 pik1 bub1 mpk2 yak103 ark1 ykl1 xks1 mak3202 mkk2 tel1 ste11 dak101
[Kinase set 4-2]
STM score
ste50 hxl1 pos5 sat4 atg1 vps15 mec1 yak1 met3 mps1 rim15 cdc7 cka1 irk4 pik1 bub1 mpk2 yak103 ark1 ykl1 xks1 mak3202 mkk2 tel1 ste11 dak101

Continued

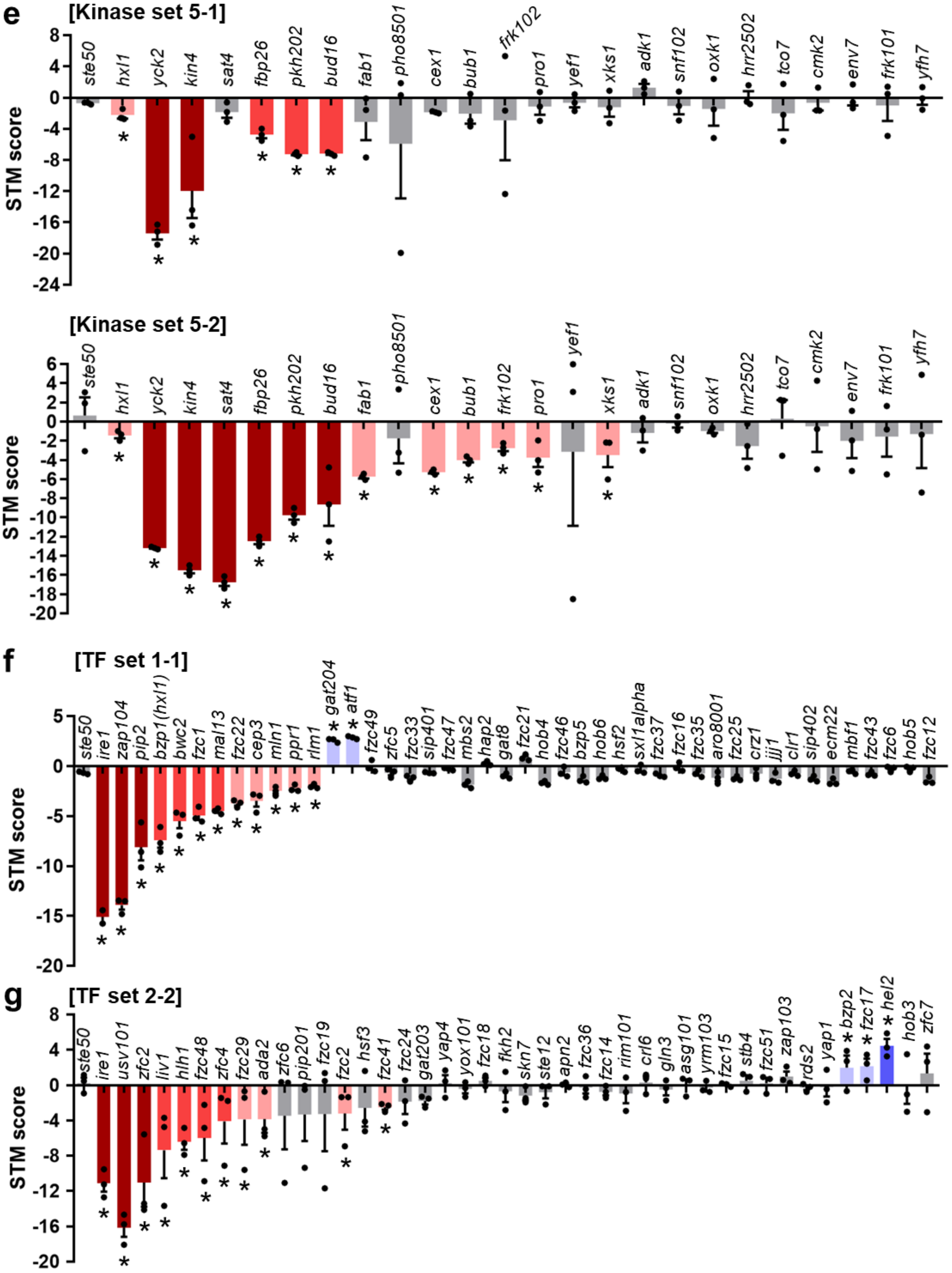
e
[Kinase set 5-1]
STM score
ste50 hxl1 yck2 kin4 sat4 fbp26 pkh202 bud16 fab1 pho8501 cex1 bub1 frk102 pro1 yef1 xks1 adk1 snf102 oxk1 hrr2502 tco7 cmk2 env7 frk101 yfh7
8 4 0 -4 -8 -12 -16 -20 -24
[Kinase set 5-2]
STM score
ste50 hxl1 yck2 kin4 sat4 fbp26 pkh202 bud16 fab1 pho8501 cex1 bub1 frk102 pro1 yef1 xks1 adk1 snf102 oxk1 hrr2502 tco7 cmk2 env7 frk101 yfh7
6 4 2 0 -2 -4 -6 -8 -10 -12 -14 -16 -18 -20
f
[TF set 1-1]
STM score
ste50 ire1 zap104 pip2 bzp1(hxl1) bwc2 fzc1 mal13 fzc22 cep3 mln1 ppr1 rlm1 gat204 atf1 fzc49 zfc5 fzc33 sip401 fzc47 mbs2 hap2 gat8 fzc21 hob4 fzc46 bzp5 hob6 hsf2 sxl1alpha fzc37 fzc16 fzc35 aro8001 fzc25 crz1 jjj1 clr1 sip402 ecm22 mbf1 fzc43 fzc6 hob5 fzc12
5 0 -5 -10 -15 -20
g
[TF set 2-2]
STM score
ste50 ire1 usv101 zfc2 liv1 hlh1 fzc48 zfc4 fzc29 ada2 zfc6 pip201 fzc19 fzc2 hsf3 fzc41 fzc24 gat203 yap4 yox101 fzc18 fkh2 skn7 ste12 apn2 fzc36 fzc14 rim101 crl6 gln3 asg101 yrm103 fzc15 stb4 fzc51 zap103 rds2 yap1 bzp2 fzc17 hel2 hob3 zfc7
8 4 0 -4 -8 -12 -16 -20

Continued

h [TF set 3-2]

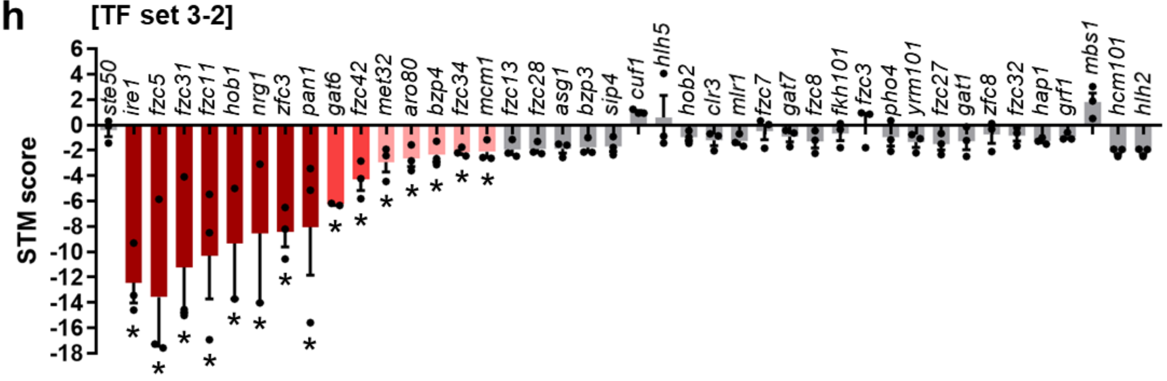

i [TF set 4-2]

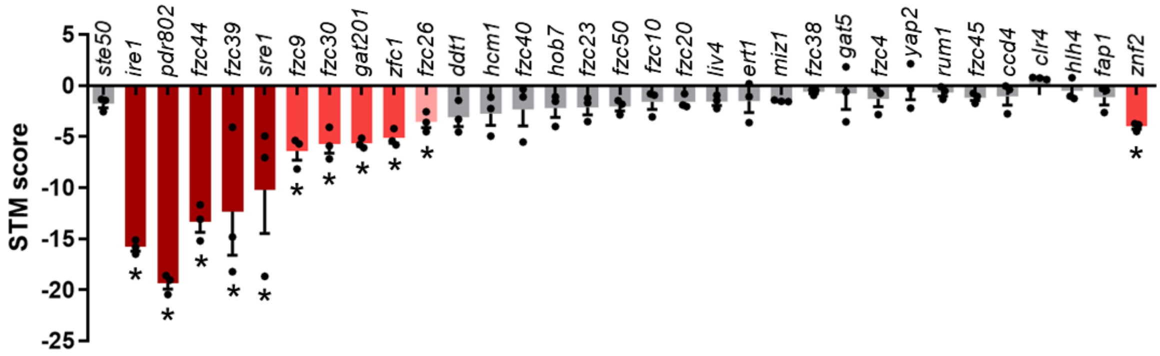

Supplementary Figure 1. Brain-STM scores in a murine model infected through intranasal instillation of kinase and TF mutant libraries. Brain STM scores were obtained from brain samples recovered from A/J mice that were intranasally infected with TF and kinase mutant libraries in our previous reports[1,2]. The STM scores of kinase (**a-e**) and TF (**f-i**) mutants of *C. neoformans* were measured by using quantitative PCR analysis with a common primer and signature-tag specific primer pairs as previously described[1,2]. Three mice were analysed in each set (n=3). Data are presented as mean values ± standard error of the mean (SEM). The STM cutoff was >2.0 (high) or <−2.0 (low) and *P*<0.05 (*) by one-way ANOVA analysis with Bonferroni's correction was considered to be statistically significant. Degrees of statistically significant increase and decrease in STM scores were indicated with different colour codes: pink (<−2), reddish (<−4), red (<−8), light blue (>2), and blue (>4). In this nasal instillation model, some of the mouse brain samples did not result in sufficient amount of *C. neoformans* cells to perform brain STM analysis. In that case, STM scores obtained from one or two mouse brain samples was used.

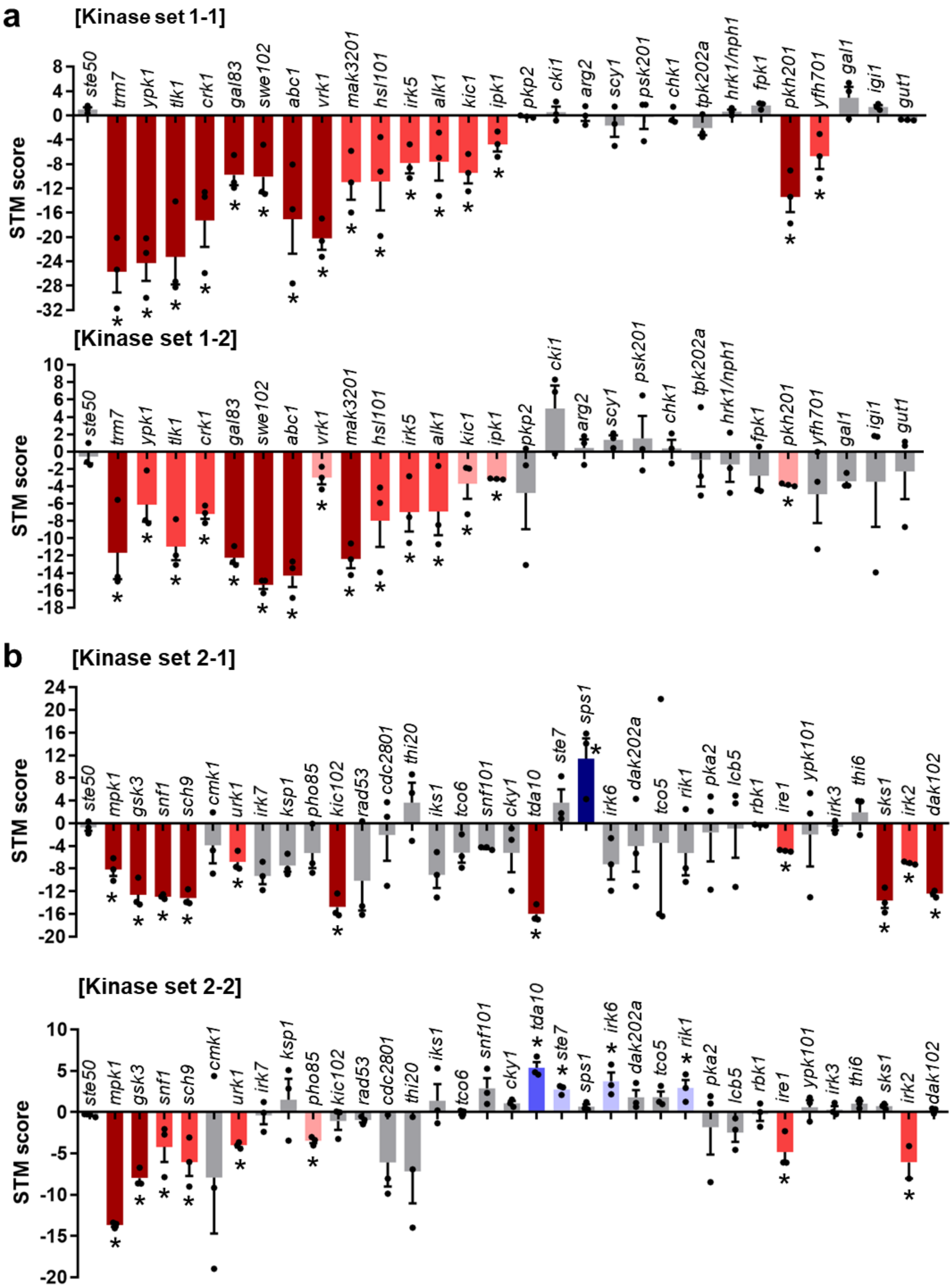
a
[Kinase set 1-1]
STM score
ste50 trm7 ypk1 tlk1 crk1 gal83 swe102 abc1 vrk1 mak3201 hsl101 irk5 alk1 kic1 ipk1 pkp2 cki1 arg2 scy1 psk201 chk1 tpk202a hrk1/nph1 fpk1 pkh201 yfh701 gal1 igi1 gut1
[Kinase set 1-2]
STM score
ste50 trm7 ypk1 tlk1 crk1 gal83 swe102 abc1 vrk1 mak3201 hsl101 irk5 alk1 kic1 ipk1 pkp2 cki1 arg2 scy1 psk201 chk1 tpk202a hrk1/nph1 fpk1 pkh201 yfh701 gal1 igi1 gut1
b
[Kinase set 2-1]
STM score
ste50 mpk1 gsk3 snf1 sch9 cmk1 urk1 irk7 ksp1 pho85 kic102 rad53 cdc2801 thi20 iks1 tco6 snf101 cky1 tda10 ste7 sps1 irk6 dak202a tco5 rik1 pka2 lcb5 rbk1 ire1 ypk101 irk3 thi6 sks1 irk2 dak102
[Kinase set 2-2]
STM score
ste50 mpk1 gsk3 snf1 sch9 cmk1 urk1 irk7 ksp1 pho85 kic102 rad53 cdc2801 thi20 iks1 tco6 snf101 cky1 tda10 ste7 sps1 irk6 dak202a tco5 rik1 pka2 lcb5 rbk1 ire1 ypk101 irk3 thi6 sks1 irk2 dak102

Continued

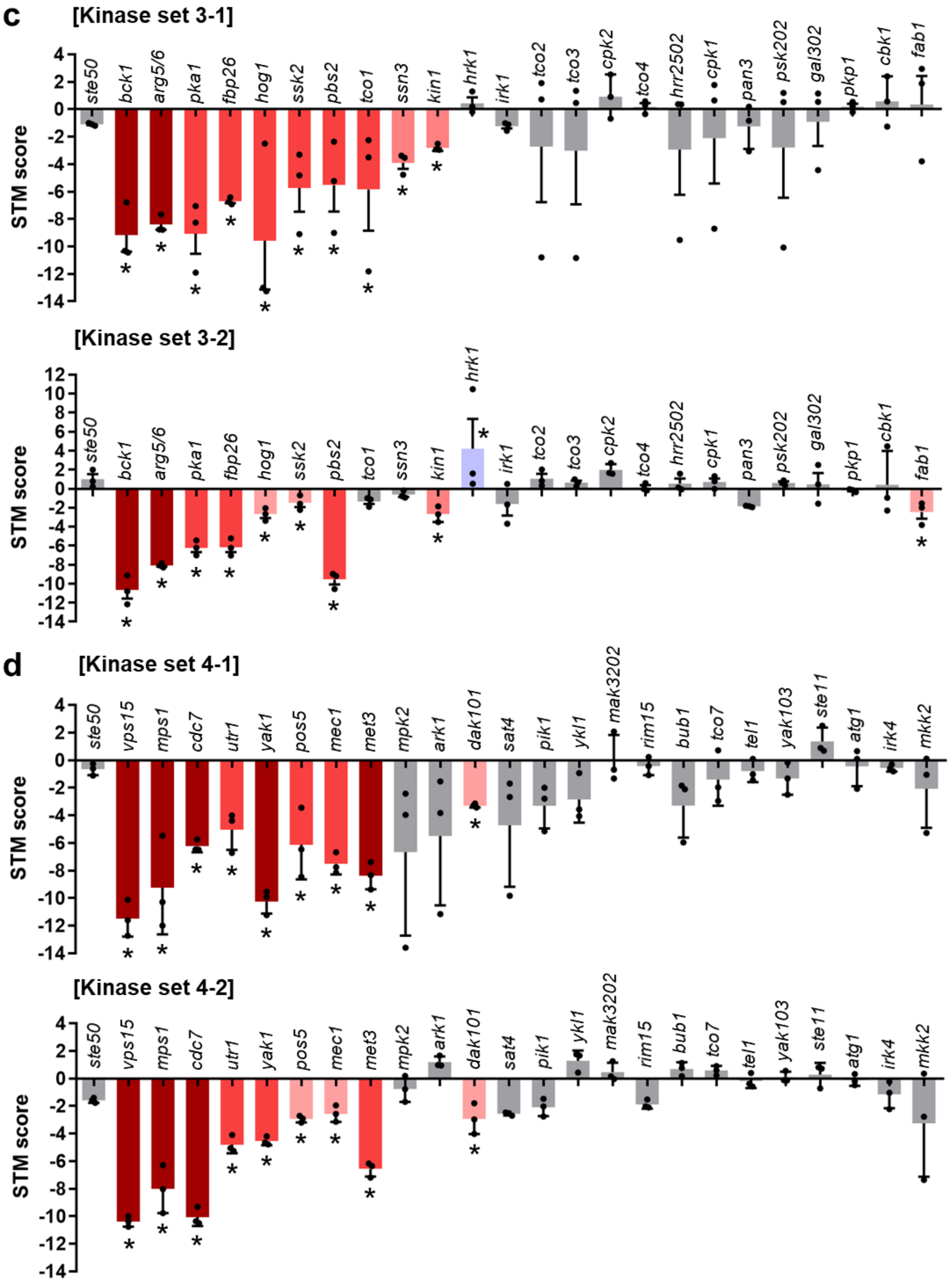
c
[Kinase set 3-1]
STM score
ste50 bck1 arg5/6 pka1 fbp26 hog1 ssk2 pbs2 tco1 ssn3 kin1 hrk1 irk1 tco2 tco3 cpk2 tco4 hrr2502 cpk1 pan3 psk202 gal302 pkp1 cbk1 fab1
[Kinase set 3-2]
STM score
ste50 bck1 arg5/6 pka1 fbp26 hog1 ssk2 pbs2 tco1 ssn3 kin1 hrk1 irk1 tco2 tco3 cpk2 tco4 hrr2502 cpk1 pan3 psk202 gal302 pkp1 cbk1 fab1
d
[Kinase set 4-1]
STM score
ste50 vps15 mps1 cdc7 utr1 yak1 pos5 mec1 met3 mpk2 ark1 dak101 sat4 pik1 ykl1 mak3202 rim15 bub1 tco7 tel1 yak103 ste11 atg1 irk4 mkk2
[Kinase set 4-2]
STM score
ste50 vps15 mps1 cdc7 utr1 yak1 pos5 mec1 met3 mpk2 ark1 dak101 sat4 pik1 ykl1 mak3202 rim15 bub1 tco7 tel1 yak103 ste11 atg1 irk4 mkk2

Continued

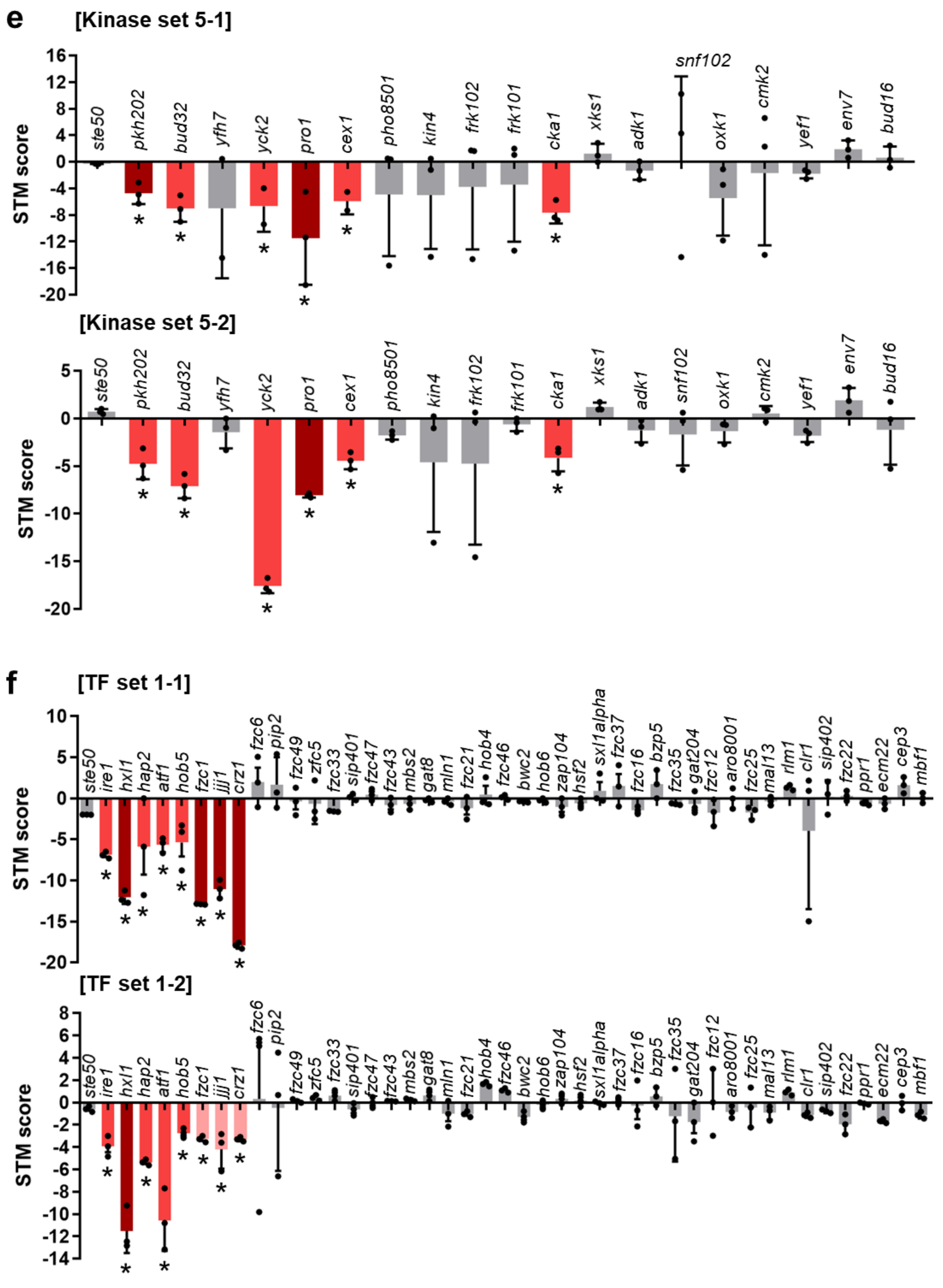
e
[Kinase set 5-1]
STM score
16
12
8
4
0
-4
-8
-12
-16
-20
ste50
pkh202
bud32
yfh7
yck2
pro1
cex1
pho8501
kin4
frk102
frk101
cka1
xks1
adk1
snf102
oxk1
cmk2
yef1
env7
bud16
*
[Kinase set 5-2]
STM score
5
0
-5
-10
-15
-20
ste50
pkh202
bud32
yfh7
yck2
pro1
cex1
pho8501
kin4
frk102
frk101
cka1
xks1
adk1
snf102
oxk1
cmk2
yef1
env7
bud16
f
[TF set 1-1]
STM score
10
5
0
-5
-10
-15
-20
ste50
ire1
hxl1
hap2
atf1
hob5
fzc1
jjj1
crz1
fzc6
pip2
fzc49
zfc5
fzc33
sip401
fzc47
fzc43
mbs2
gat8
mln1
fzc21
hob4
fzc46
bwc2
hob6
zap104
hsf2
sxl1alpha
fzc37
fzc16
bzp5
fzc35
gat204
fzc12
aro8001
fzc25
mal13
rlm1
clr1
sip402
fzc22
ppr1
ecm22
cep3
mbf1
[TF set 1-2]
STM score
8
6
4
2
0
-2
-4
-6
-8
-10
-12
-14

Continued

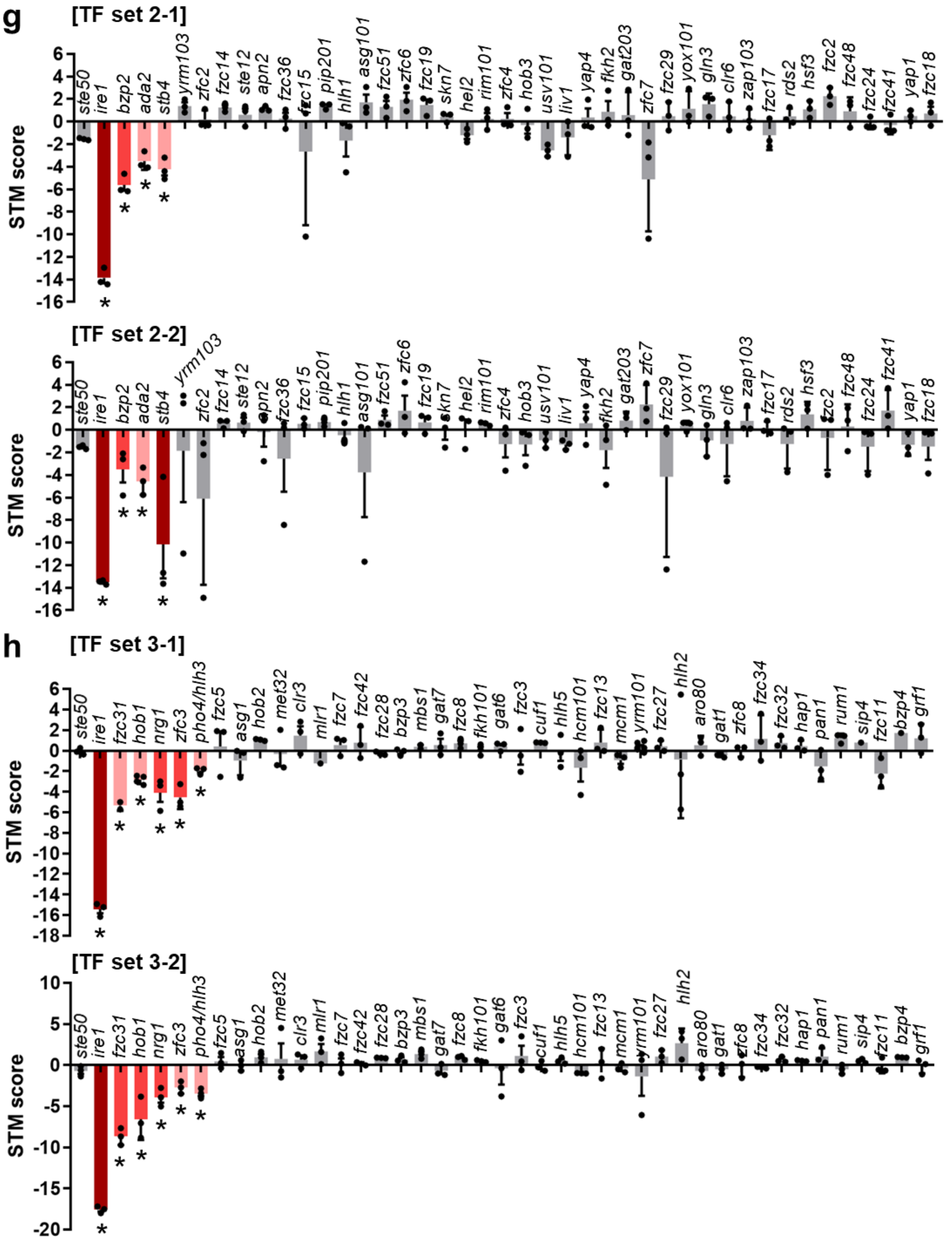
g
[TF set 2-1]
STM score
6 4 2 0 -2 -4 -6 -8 -10 -12 -14 -16
ste50 ire1 bzp2 ada2 stb4 yrm103 zfc2 fzc14 ste12 apn2 fzc36 fzc15 pip201 hlh1 asg101 fzc51 zfc6 fzc19 skn7 hel2 rim101 zfc4 hob3 usv101 liv1 yap4 fkh2 gat203 zfc7 fzc29 yox101 gln3 clr6 zap103 fzc17 rds2 hsf3 fzc2 fzc48 fzc24 fzc41 yap1 fzc18
[TF set 2-2]
STM score
6 4 2 0 -2 -4 -6 -8 -10 -12 -14 -16
ste50 ire1 bzp2 ada2 stb4 yrm103 zfc2 fzc14 ste12 apn2 fzc36 fzc15 pip201 hlh1 asg101 fzc51 zfc6 fzc19 skn7 hel2 rim101 zfc4 hob3 usv101 liv1 yap4 fkh2 gat203 zfc7 fzc29 yox101 gln3 clr6 zap103 fzc17 rds2 hsf3 fzc2 fzc48 fzc24 fzc41 yap1 fzc18
h
[TF set 3-1]
STM score
6 4 2 0 -2 -4 -6 -8 -10 -12 -14 -16 -18
ste50 ire1 fzc31 hob1 nrg1 zfc3 pho4/hlh3 fzc5 asg1 hob2 met32 clr3 mlr1 fzc7 fzc42 fzc28 bzp3 mbs1 gat7 fzc8 fkh101 gat6 fzc3 cuf1 hlh5 hcm101 fzc13 mcm1 yrm101 fzc27 hlh2 aro80 gat1 zfc8 fzc34 fzc32 hap1 pan1 rum1 sip4 fzc11 bzp4 grf1
[TF set 3-2]
STM score
10 5 0 -5 -10 -15 -20
ste50 ire1 fzc31 hob1 nrg1 zfc3 pho4/hlh3 fzc5 asg1 hob2 met32 clr3 mlr1 fzc7 fzc42 fzc28 bzp3 mbs1 gat7 fzc8 fkh101 gat6 fzc3 cuf1 hlh5 hcm101 fzc13 mcm1 yrm101 fzc27 hlh2 aro80 gat1 zfc8 fzc34 fzc32 hap1 pan1 rum1 sip4 fzc11 bzp4 grf1

Continued

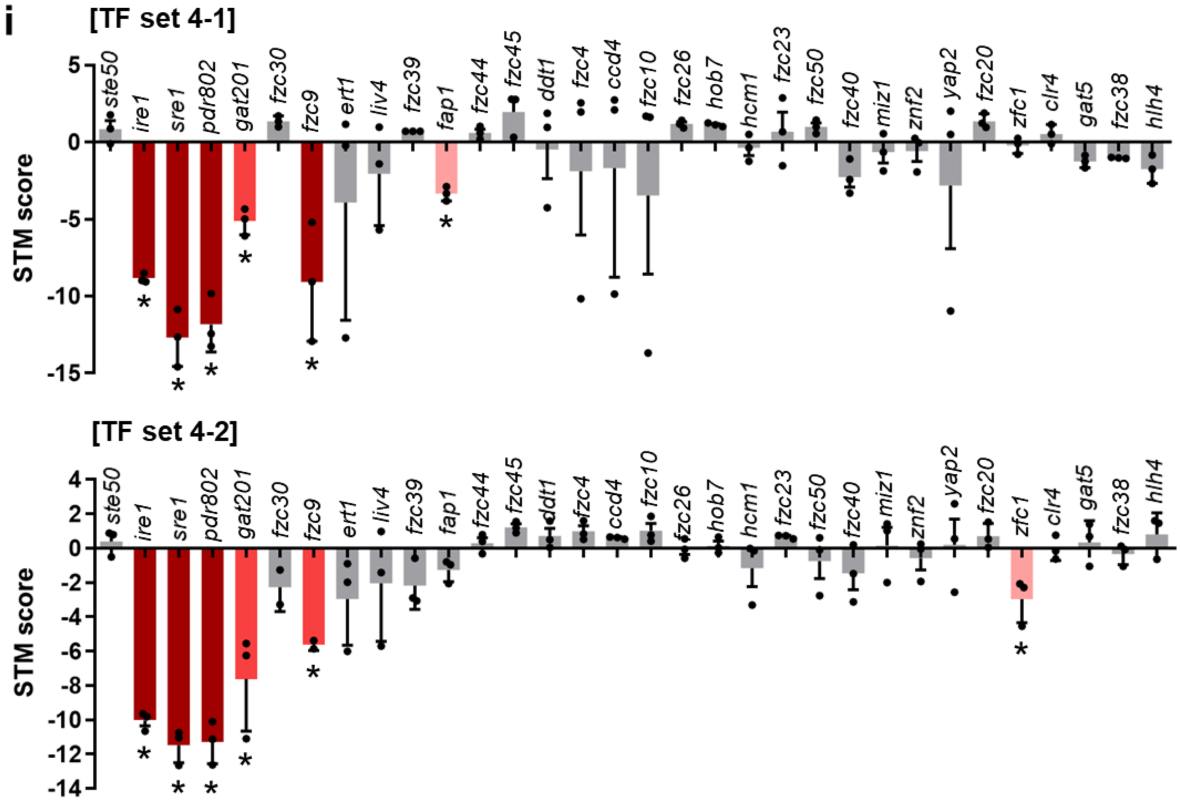

Supplementary Figure 2. Brain-STM scores in a murine model infected through intravenous injection of kinase and TF mutant libraries. Brain STM scores were obtained from brain samples recovered from A/J mice that were intravenously infected in this study with TF and kinase mutant libraries[1,2]. STM scores of kinase (**a**-**e**) and TF (**f**-**i**) mutants of *C. neoformans*, which are listed in Supplementary Table 3, were measured by using quantitative PCR analysis with a common primer and signature-tag specific primer pairs as previously described[1,2]. Dash 1 and dash 2 of each set indicate data for two-independent mutant strains for each gene (Supplementary Table 3). Three mice were analysed in each set (n=3). Data are presented as mean values ± SEM. The STM cutoff was >2.0 (high) or <−2.0 (low) and *P*<0.05 (*) by one-way ANOVA analysis with Bonferroni's correction was considered to be statistically significant. Degrees of statistically significant increase and decrease in STM scores were indicated with different color codes: pink (<−2), reddish (<−4), red (<−8), light blue (>2), and blue (>4).

a

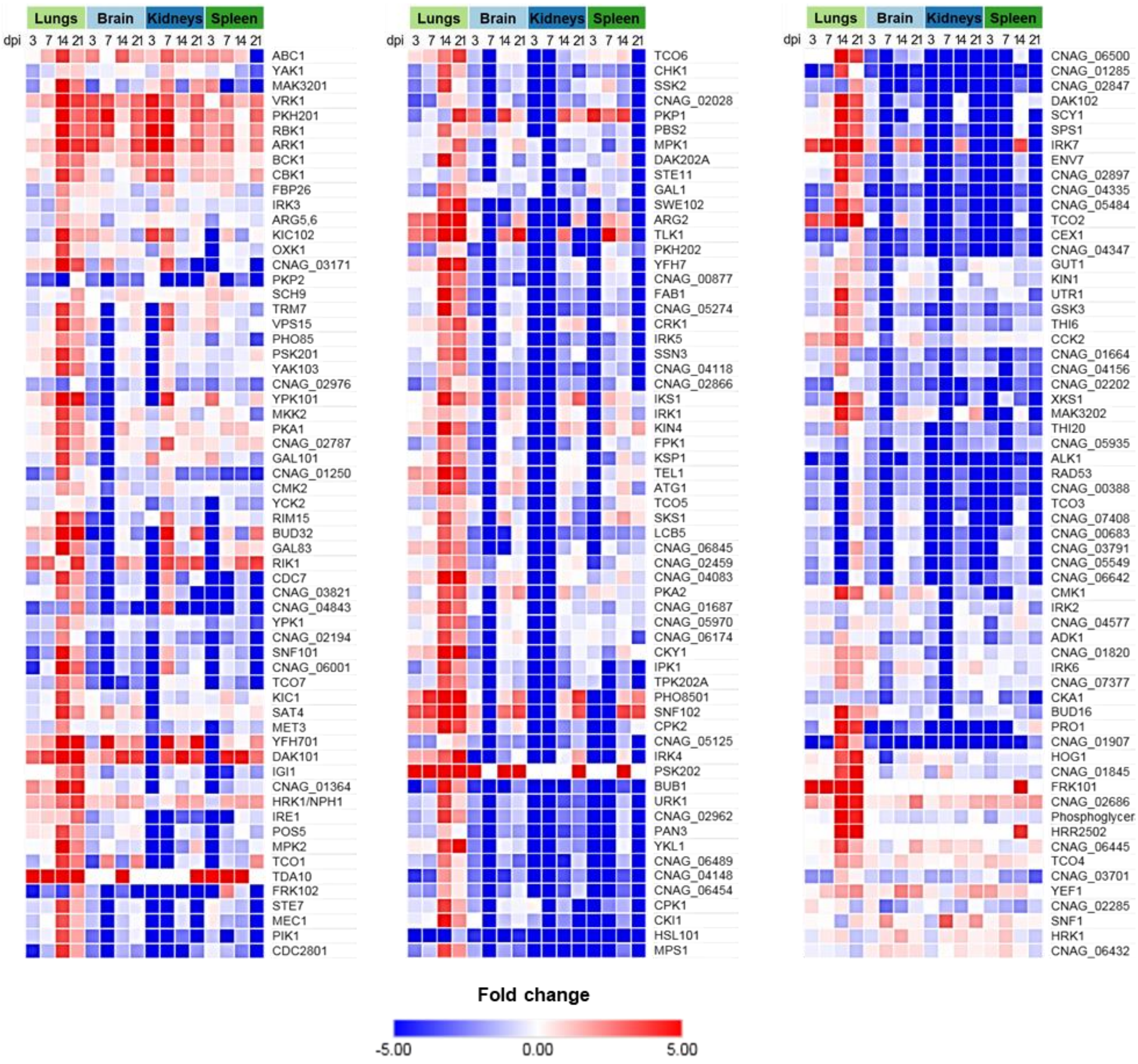
Lungs
Brain
Kidneys
Spleen
dpi 3 7 14 21 3 7 14 21 3 7 14 21 3 7 14 21
ABC1
YAK1
MAK3201
VRK1
PKH201
RBK1
ARK1
BCK1
CBK1
FBP26
IRK3
ARG5,6
KIC102
OXK1
CNAG_03171
PKP2
SCH9
TRM7
VPS15
PHO85
PSK201
YAK103
CNAG_02976
YPK101
MKK2
PKA1
CNAG_02787
GAL101
CNAG_01250
CMK2
YCK2
RIM15
BUD32
GAL83
RIK1
CDC7
CNAG_03821
CNAG_04843
YPK1
CNAG_02194
SNF101
CNAG_06001
TCO7
KIC1
SAT4
MET3
YFH701
DAK101
IGI1
CNAG_01364
HRK1/NPH1
IRE1
POS5
MPK2
TCO1
TDA10
FRK102
STE7
MEC1
PIK1
CDC2801
TCO6
CHK1
SSK2
CNAG_02028
PKP1
PBS2
MPK1
DAK202A
STE11
GAL1
SWE102
ARG2
TLK1
PKH202
YFH7
CNAG_00877
FAB1
CNAG_05274
CRK1
IRK5
SSN3
CNAG_04118
CNAG_02866
IKS1
IRK1
KIN4
FPK1
KSP1
TEL1
ATG1
TCO5
SKS1
LCB5
CNAG_06845
CNAG_02459
CNAG_04083
PKA2
CNAG_01687
CNAG_05970
CNAG_06174
CKY1
IPK1
TPK202A
PHO8501
SNF102
CPK2
CNAG_05125
IRK4
PSK202
BUB1
URK1
CNAG_02962
PAN3
YKL1
CNAG_06489
CNAG_04148
CNAG_06454
CPK1
CKI1
HSL101
MPS1
CNAG_06500
CNAG_01285
CNAG_02847
DAK102
SCY1
SPS1
IRK7
ENV7
CNAG_02897
CNAG_04335
CNAG_05484
TCO2
CEX1
CNAG_04347
GUT1
KIN1
UTR1
GSK3
THI6
CCK2
CNAG_01664
CNAG_04156
CNAG_02202
XKS1
MAK3202
THI20
CNAG_05935
ALK1
RAD53
CNAG_00388
TCO3
CNAG_07408
CNAG_00683
CNAG_03791
CNAG_05549
CNAG_06642
CMK1
IRK2
CNAG_04577
ADK1
CNAG_01820
IRK6
CNAG_07377
CKA1
BUD16
PRO1
CNAG_01907
HOG1
CNAG_01845
FRK101
CNAG_02686
Phosphoglycer
HRR2502
CNAG_06445
TCO4
CNAG_03701
YEF1
CNAG_02285
SNF1
HRK1
CNAG_06432
Fold change
-5.00
0.00
5.00

Continued

b

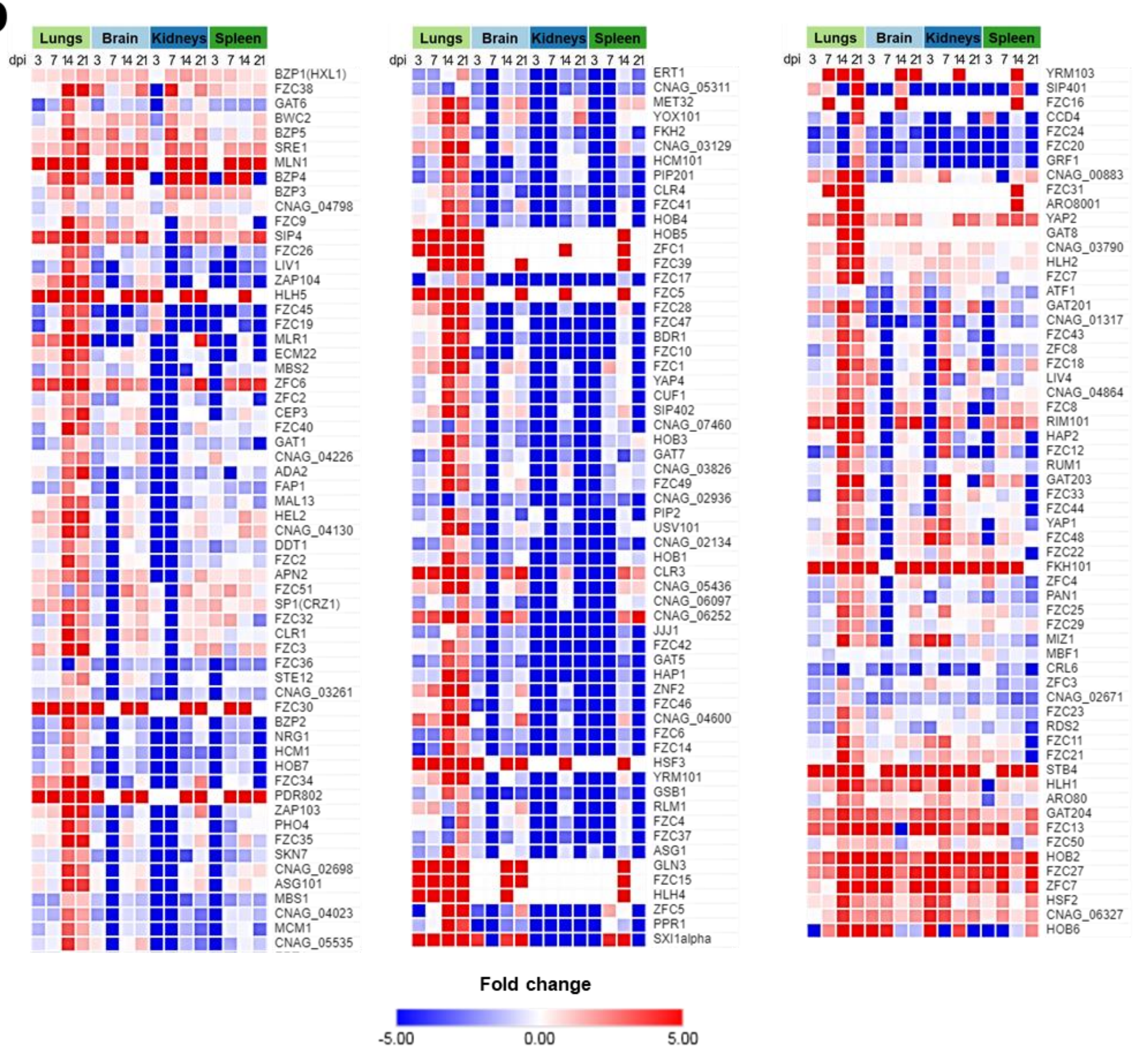

Supplementary Figure 3. In vivo gene expression patterns of kinases and TFs in *C. neoformans.* NanoString^{TM}-nCounter$^{®}$ based in vivo gene expression analysis of 183 kinases (**a**) and 180 TFs (**b**) was performed using total RNA extracted from the infected tissues (lungs, brain, spleen, and kidneys) recovered after 3, 7, 14, and 21 dpi from a murine cryptococcosis model by intranasal instillation and gene-specific nCounter codesets (Supplementary Data 1). Fold-change heatmap was clustered using one minus Pearson correlation with average linkage by Morpheus. Each in vivo gene expression level was normalised by that of 8 house-keeping genes in the same tissue sample. Three A/J mice per dpi cohort were used (total 12 mice).

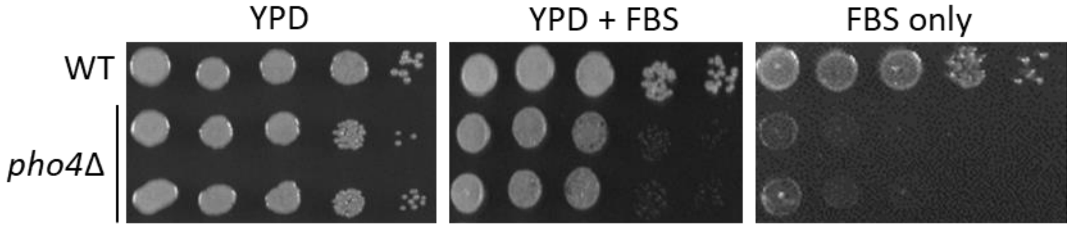

Supplementary Figure 4. Serum sensitivity assay of *pho4*Δ mutants. *Cryptococcus neoformans* WT (H99) and *pho4*Δ mutants (YSB2329 and YSB2330) were cultured overnight in yeast extract-peptone-dextrose (YPD) medium and spotted onto YPD, YPD+FBS (YPD with 50% fetal bovine serum), or FBS only (FBS plate, final 6.25% FBS). For the FBS supplementation, FBS was added into autoclaved 2× YPD agar or 10% agar. Plates were photographed for 3 days. This spot assay was repeated twice, and one representative image was shown here.

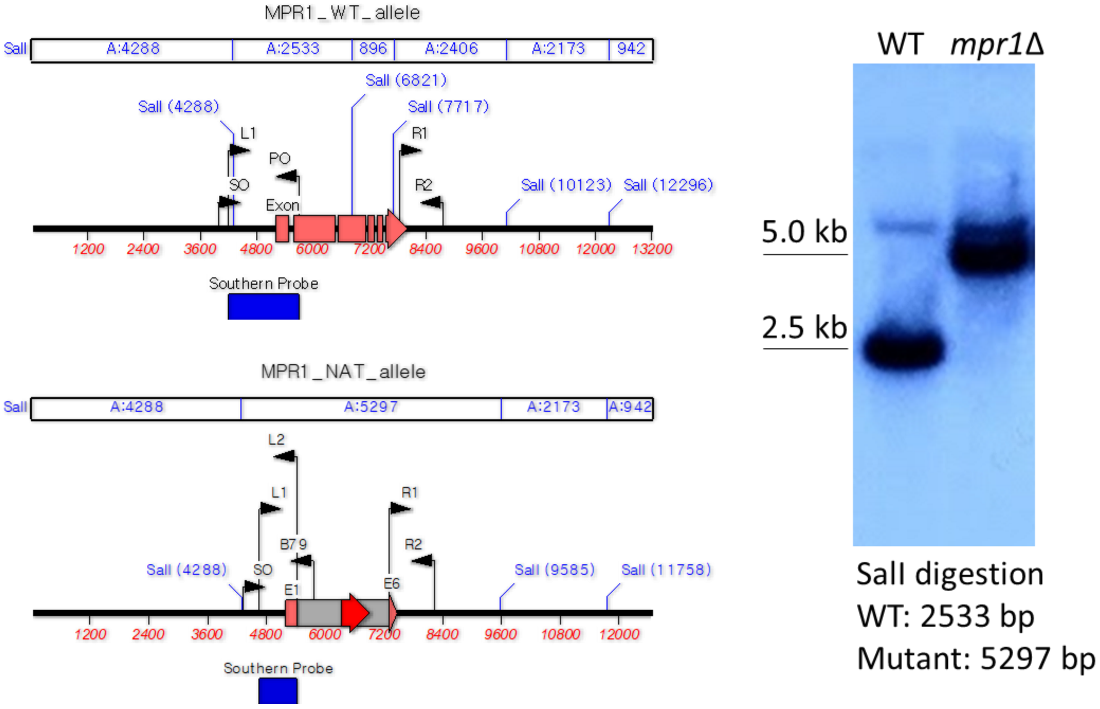

Supplementary Figure 5. Construction of the *mpr1*Δ mutant in *C. neoformans*. The diagram illustrates *MPR1* gene disruption strategy by showing primer binding and enzyme cut sites in WT and *mpr1*Δ mutant strains. Primers used for amplification of the 5′- and 3′-flanking regions, overlap PCR for NAT^r-split marker, diagnostic PCR for mutant screening, and Southern blot probe are listed in Supplementary Table 4. SalI digested genomic DNA of WT and *mpr1*Δ (YSB5492) was used for Southern blot analysis. The Southern blot analysis was repeated twice and one representative image was shown here. Source data are provided as a Source Data file.

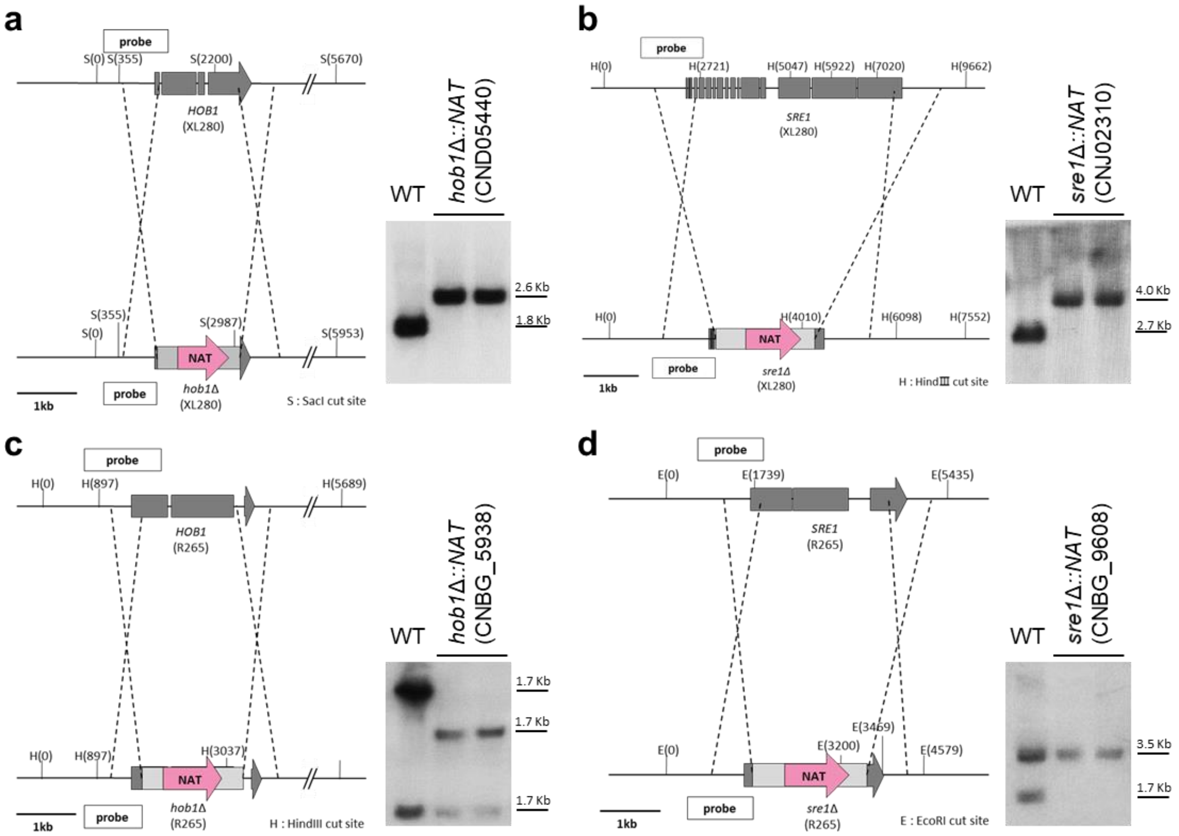

Supplementary Figure 6. Disruption of *HOB1* and *SRE1* genes in XL280 and R265 strain backgrounds. (**a**) Diagram for disruption of the *HOB1* gene in XL280 strain by overlap-PCR with *NAT*^{r}-split marker. The correct gene disruption was verified by Southern blot analysis using genomic DNA digested with SacI restriction enzyme. (**b**) Diagram for disruption of the *SRE1* gene in XL280 strain by overlap-PCR with *NAT*^{r}-split marker. The correct gene disruption was verified by Southern blot analysis using genomic DNA digested with HindIII restriction enzyme. (**c**) Diagram for disruption of the *HOB1* gene in R265 strain by overlap-PCR with *NAT*^{r}-split marker. The correct gene disruption was verified by Southern blot analysis using genomic DNA digested with HindIII restriction enzyme. (**d**) Diagram for disruption of the *SRE1* gene in R265 strain by overlap-PCR with *NAT*^{r}-split marker. The correct gene disruption was verified by Southern blot analysis using genomic DNA digested with EcoRI restriction enzyme. Each gene disruption cassette was introduced into each strain by biolistic transformation. All Southern blot analyses (**a-d**) were repeated twice and each representative image was shown here. Source data are provided as a Source Data file.

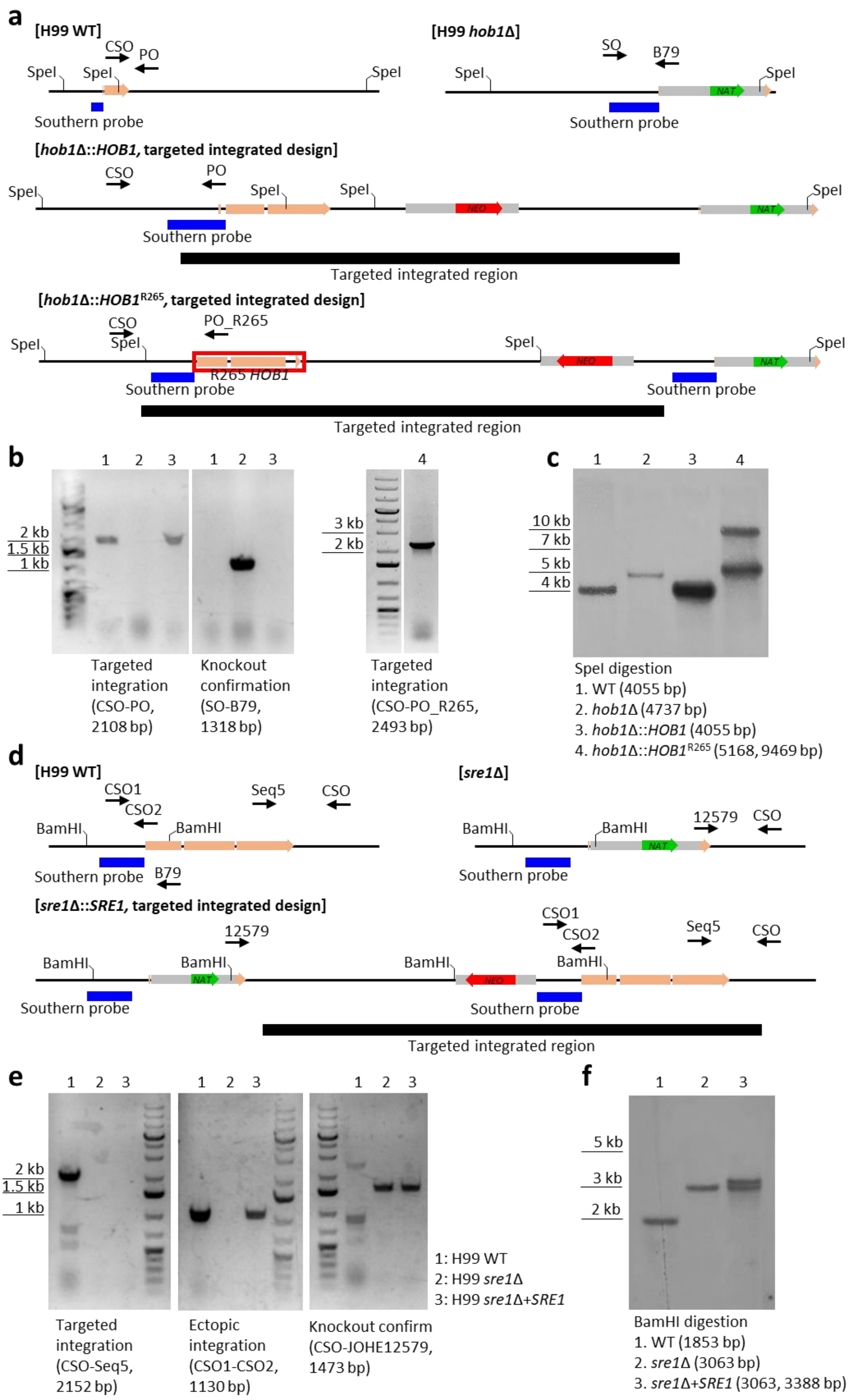
a
[H99 WT]
CSO
PO
SpeI
SpeI
SpeI
Southern probe
[H99 hob1Δ]
SO
B79
SpeI
NAT
SpeI
Southern probe
[hob1Δ::HOB1, targeted integrated design]
CSO
PO
SpeI
SpeI
SpeI
NEO
NAT
SpeI
Southern probe
Targeted integrated region
[hob1Δ::HOB1R265, targeted integrated design]
CSO
PO_R265
SpeI
SpeI
SpeI
NEO
NAT
SpeI
R265 HOB1
Southern probe
Southern probe
Targeted integrated region
b
1 2 3 1 2 3
4
2 kb
1.5 kb
1 kb
3 kb
2 kb
Targeted integration (CSO-PO, 2108 bp)
Knockout confirmation (SO-B79, 1318 bp)
Targeted integration (CSO-PO_R265, 2493 bp)
c
1 2 3 4
10 kb
7 kb
5 kb
4 kb
SpeI digestion
1. WT (4055 bp)
2. hob1Δ (4737 bp)
3. hob1Δ::HOB1 (4055 bp)
4. hob1Δ::HOB1R265 (5168, 9469 bp)
d
[H99 WT]
CSO1
CSO2
Seq5
CSO
BamHI
BamHI
Southern probe
B79
[sre1Δ]
BamHI
BamHI
12579
CSO
NAT
Southern probe
[sre1Δ::SRE1, targeted integrated design]
12579
BamHI
BamHI
NAT
CSO1
CSO2
Seq5
CSO
BamHI
NEO
BamHI
Southern probe
Southern probe
Targeted integrated region
e
1 2 3
1 2 3
1 2 3
2 kb
1.5 kb
1 kb
Targeted integration (CSO-Seq5, 2152 bp)
Ectopic integration (CSO1-CSO2, 1130 bp)
Knockout confirm (CSO-JOHE12579, 1473 bp)
1: H99 WT
2: H99 sre1Δ
3: H99 sre1Δ+SRE1
f
1 2 3
5 kb
3 kb
2 kb
BamHI digestion
1. WT (1853 bp)
2. sre1Δ (3063 bp)
3. sre1Δ+SRE1 (3063, 3388 bp)

Continued

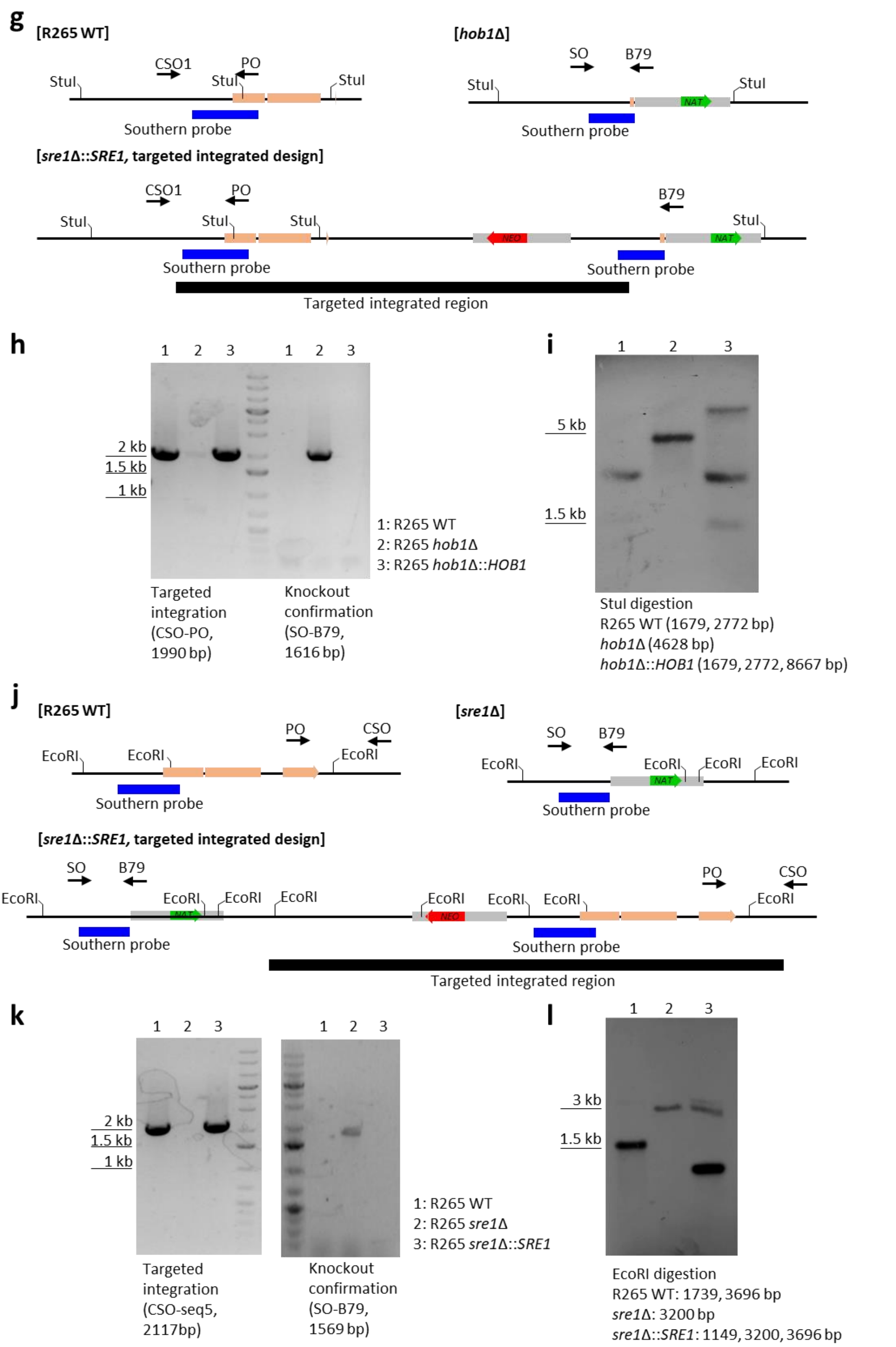
g
[R265 WT]
CSO1
PO
StuI
StuI
StuI
Southern probe
[hob1Δ]
SO
B79
StuI
StuI
NAT
Southern probe
[sre1Δ::SRE1, targeted integrated design]
CSO1
PO
B79
StuI
StuI
StuI
StuI
NEO
NAT
Southern probe
Southern probe
Targeted integrated region
h
1 2 3 1 2 3
2 kb
1.5 kb
1 kb
1: R265 WT
2: R265 hob1Δ
3: R265 hob1Δ::HOB1
Targeted integration (CSO-PO, 1990 bp)
Knockout confirmation (SO-B79, 1616 bp)
i
1 2 3
5 kb
1.5 kb
StuI digestion
R265 WT (1679, 2772 bp)
hob1Δ (4628 bp)
hob1Δ::HOB1 (1679, 2772, 8667 bp)
j
[R265 WT]
PO
CSO
EcoRI
EcoRI
EcoRI
Southern probe
[sre1Δ]
SO
B79
EcoRI
EcoRI
EcoRI
EcoRI
NAT
Southern probe
[sre1Δ::SRE1, targeted integrated design]
SO
B79
PO
CSO
EcoRI
EcoRI
EcoRI
EcoRI
EcoRI
EcoRI
EcoRI
EcoRI
NAT
NEO
Southern probe
Southern probe
Targeted integrated region
k
1 2 3 1 2 3
2 kb
1.5 kb
1 kb
1: R265 WT
2: R265 sre1Δ
3: R265 sre1Δ::SRE1
Targeted integration (CSO-seq5, 2117bp)
Knockout confirmation (SO-B79, 1569 bp)
l
1 2 3
3 kb
1.5 kb
EcoRI digestion
R265 WT: 1739, 3696 bp
sre1Δ: 3200 bp
sre1Δ::SRE1: 1149, 3200, 3696 bp

Continued

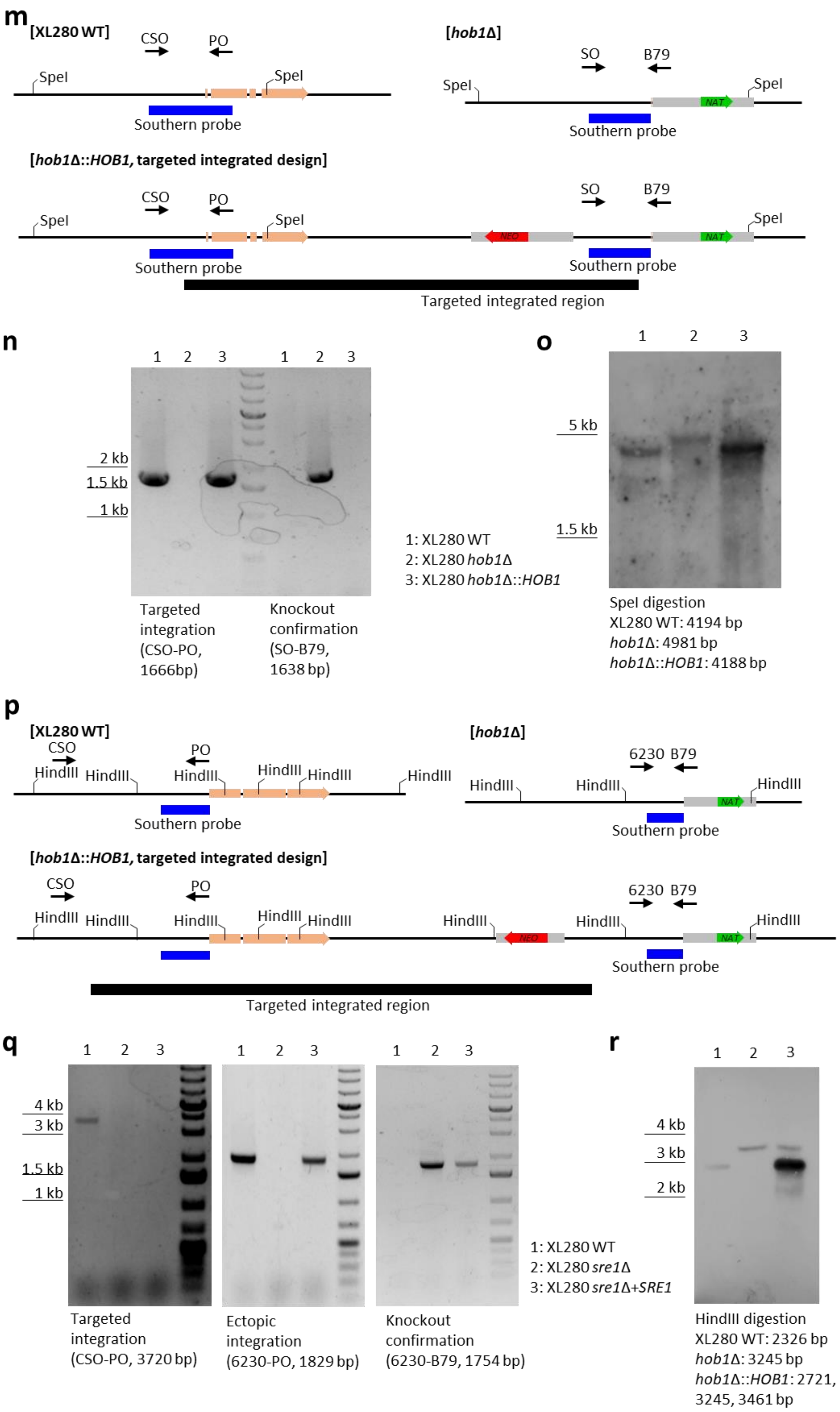
m
[XL280 WT]
CSO
PO
SpeI
SpeI
Southern probe
[hob1Δ]
SO
B79
SpeI
NAT
SpeI
Southern probe
[hob1Δ::HOB1, targeted integrated design]
CSO
PO
SpeI
SpeI
NEO
SO
B79
NAT
SpeI
Southern probe
Southern probe
Targeted integrated region
n
1 2 3 1 2 3
2 kb
1.5 kb
1 kb
Targeted integration (CSO-PO, 1666bp)
Knockout confirmation (SO-B79, 1638 bp)
1: XL280 WT
2: XL280 hob1Δ
3: XL280 hob1Δ::HOB1
o
1 2 3
5 kb
1.5 kb
SpeI digestion
XL280 WT: 4194 bp
hob1Δ: 4981 bp
hob1Δ::HOB1: 4188 bp
p
[XL280 WT]
CSO
PO
HindIII HindIII
HindIII
HindIII HindIII
HindIII
Southern probe
[hob1Δ]
6230 B79
HindIII
HindIII
NAT
HindIII
Southern probe
[hob1Δ::HOB1, targeted integrated design]
CSO
PO
HindIII HindIII
HindIII
HindIII HindIII
HindIII
NEO
HindIII
6230 B79
NAT
HindIII
Southern probe
Targeted integrated region
q
1 2 3
4 kb
3 kb
1.5 kb
1 kb
Targeted integration (CSO-PO, 3720 bp)
1 2 3
Ectopic integration (6230-PO, 1829 bp)
1 2 3
Knockout confirmation (6230-B79, 1754 bp)
1: XL280 WT
2: XL280 sre1Δ
3: XL280 sre1Δ+SRE1
r
1 2 3
4 kb
3 kb
2 kb
HindIII digestion
XL280 WT: 2326 bp
hob1Δ: 3245 bp
hob1Δ::HOB1: 2721, 3245, 3461 bp

Supplementary Figure 7. Genetic complementation of *hob1*Δ and *sre1*Δ mutants constructed in H99, R265, and XL280 strain backgrounds. Knockout and complementation of *HOB1* and *SRE1* were confirmed by diagnostic PCR and Southern blot analysis. Specific primers for diagnostic PCR and Southern probes are listed in Supplementary Table 4. (**a**-**f**) *HOB1* and *SRE1* complementation in H99 *hob1*Δ and *sre1*Δ mutants. Schematic diagram of (**a**) *HOB1* and (**d**) *SRE1* in H99 WT, knockout mutant, and complemented strains. Targeted integration of *HOB1* was confirmed by (**b**) diagnostic PCR and (**c**) Southern blot analysis with SpeI digestion. Ectopic integration of *SRE1* was confirmed by (**e**) diagnostic PCR and (**f**) Southern blot analysis with BamHI digestion. (**g**-**l**) *HOB1* and *SRE1* complementation in R265 *hob1*Δ and *sre1*Δ mutants. Schematic diagram of (**g**) *HOB1* and (**j**) *SRE1* in R265 WT, knockout mutant, and complemented strains. Targeted integration of *HOB1* was confirmed by (**h**) diagnostic PCR and (**i**) Southern blot analysis with StuI digestion. Targeted integration of *SRE1* was confirmed by (**k**) diagnostic PCR and (**l**) Southern blot analysis with EcoRI digestion. (**m**-**r**) *HOB1* and *SRE1* complementation in XL280 *hob1*Δ and *sre1*Δ mutants. Schematic diagram of (**m**) *HOB1* and (**p**) *SRE1* in XL280 WT, knockout mutant, and complemented strains. Targeted integration of *HOB1* was confirmed by (**n**) diagnostic PCR and (**o**) Southern blot analysis with SpeI digestion. Ectopic integration of *SRE1* was confirmed by (**q**) diagnostic PCR and (**r**) Southern blot analysis with HindIII digestion. All diagnostic PCR (**b**, **e**, **h**, **k**, **n**, **q**) and Southern blot (**c, f, i, l, o**, **r**) analyses were repeated twice and each representative image was shown here. Source data are provided as a Source Data file.

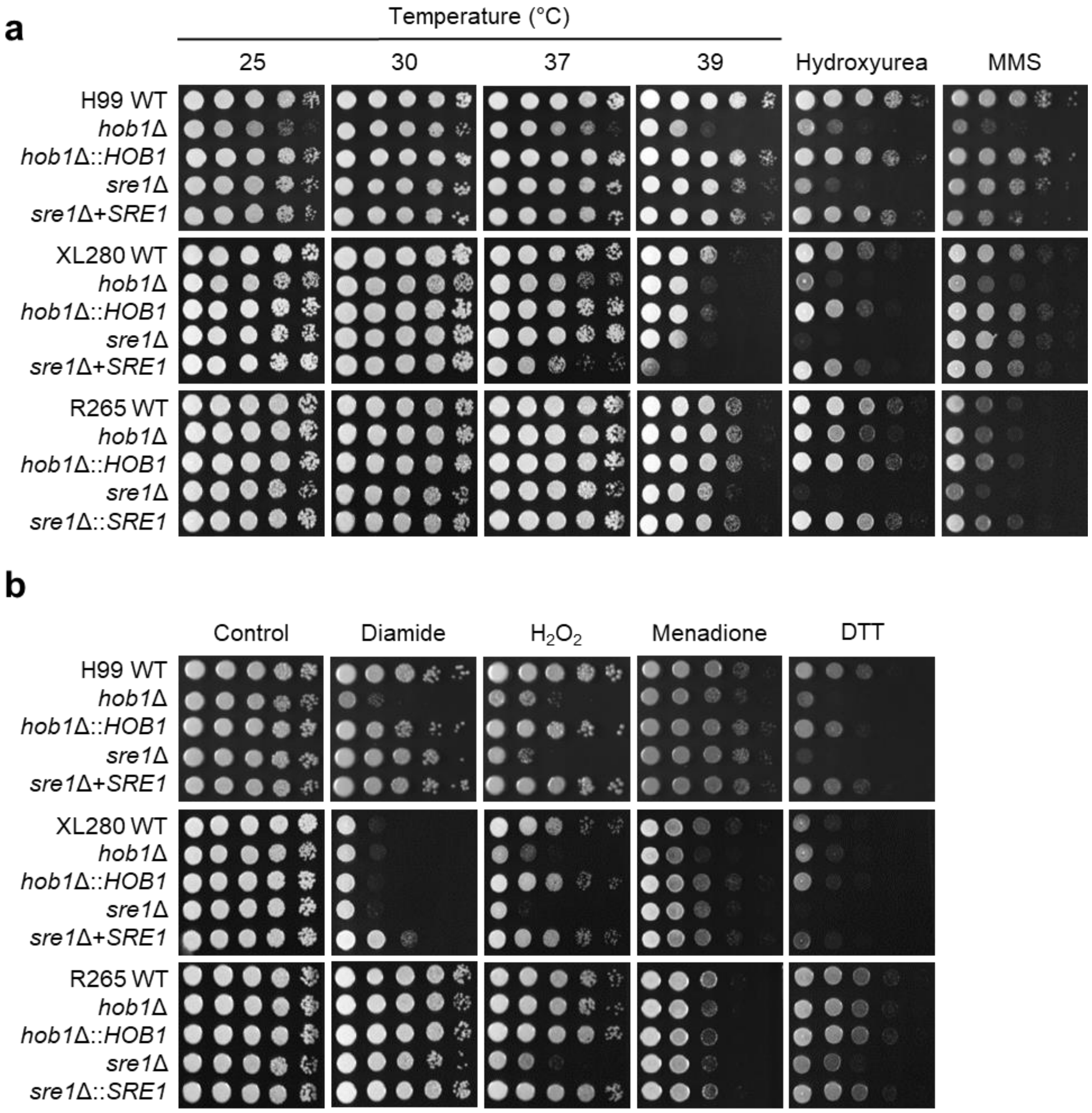
a
Temperature (°C)
25
30
37
39
Hydroxyurea
MMS
H99 WT
hob1Δ
hob1Δ::HOB1
sre1Δ
sre1Δ+SRE1
XL280 WT
hob1Δ
hob1Δ::HOB1
sre1Δ
sre1Δ+SRE1
R265 WT
hob1Δ
hob1Δ::HOB1
sre1Δ
sre1Δ::SRE1
b
Control
Diamide
H_2O_2
Menadione
DTT
H99 WT
hob1Δ
hob1Δ::HOB1
sre1Δ
sre1Δ+SRE1
XL280 WT
hob1Δ
hob1Δ::HOB1
sre1Δ
sre1Δ+SRE1
R265 WT
hob1Δ
hob1Δ::HOB1
sre1Δ
sre1Δ::SRE1

Continued

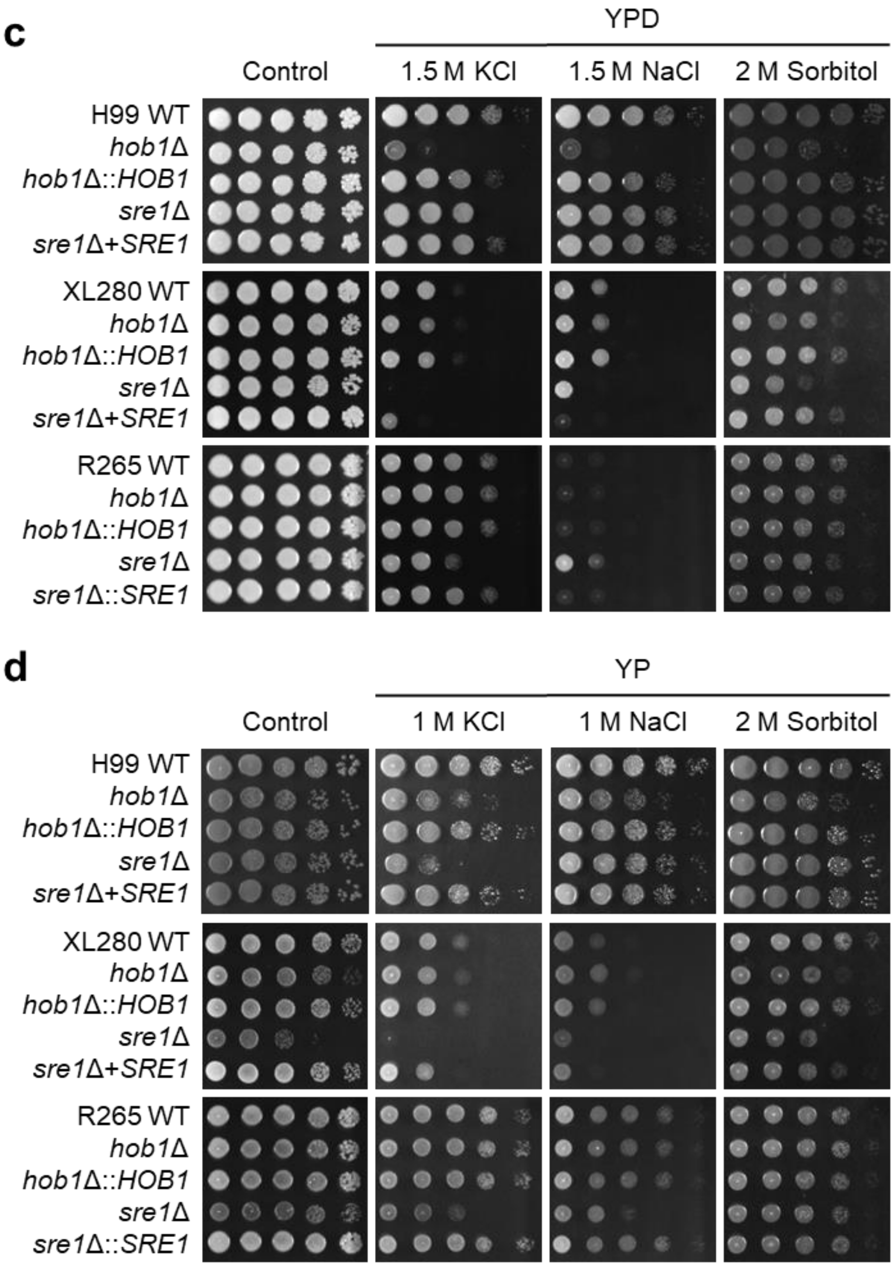
c
YPD
Control
1.5 M KCl
1.5 M NaCl
2 M Sorbitol
H99 WT
hob1Δ
hob1Δ::HOB1
sre1Δ
sre1Δ+SRE1
XL280 WT
hob1Δ
hob1Δ::HOB1
sre1Δ
sre1Δ+SRE1
R265 WT
hob1Δ
hob1Δ::HOB1
sre1Δ
sre1Δ::SRE1
d
YP
Control
1 M KCl
1 M NaCl
2 M Sorbitol
H99 WT
hob1Δ
hob1Δ::HOB1
sre1Δ
sre1Δ+SRE1
XL280 WT
hob1Δ
hob1Δ::HOB1
sre1Δ
sre1Δ+SRE1
R265 WT
hob1Δ
hob1Δ::HOB1
sre1Δ
sre1Δ::SRE1

Continued

e

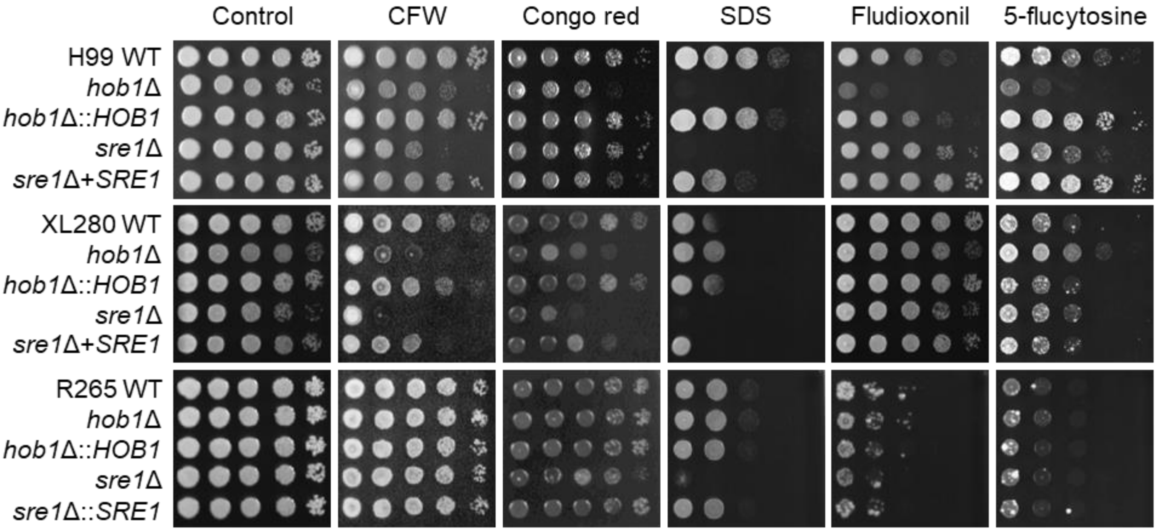

f

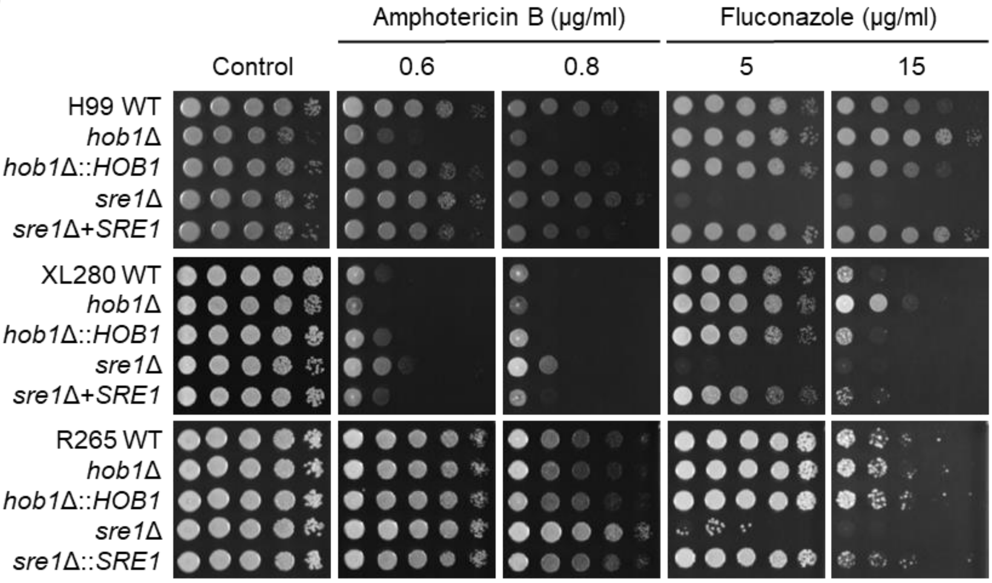

Continued

g

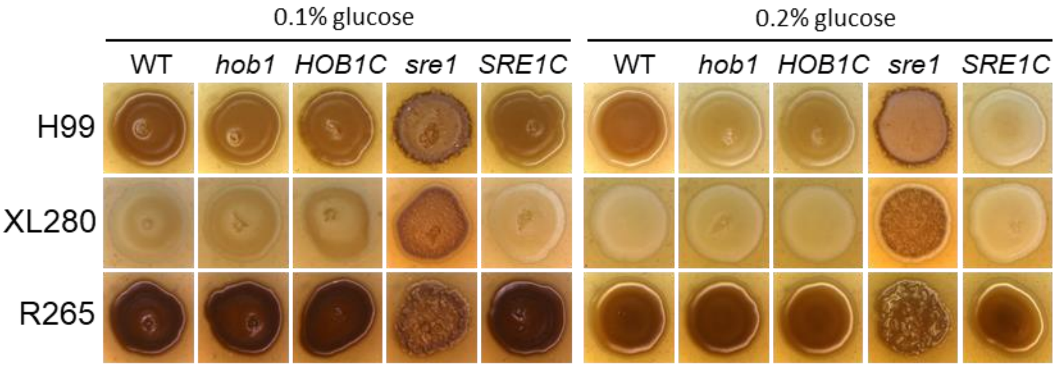

h

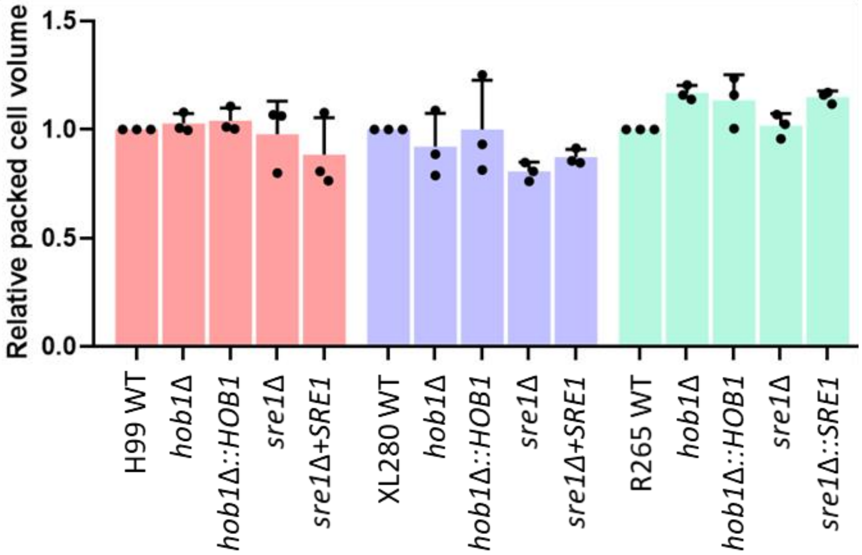

Supplementary Figure 8. Functional analysis of Hob1 and Sre1 in pathogenic *Cryptococcus* species complex. (**a-f**) The spot assay for qualitatively monitoring chemical susceptibility and thermotolerance was performed using the following WT, *hob1*Δ or *sre1*Δ mutants, and gene complemented strains: H99 strain background [*hob1*Δ (YSB2308), *hob1*Δ::*HOB1* (YSB7467), *sre1*Δ (YSB2464), *sre1*Δ+*SRE1* (YSB4847)]; XL280 strain background [*hob1*Δ (YSB3690), *hob1*Δ::*HOB1* (YSB4576), *sre1*Δ (YSB3940), *sre1*Δ::*SRE1* (YSB4904)]; R265 strain background [*hob1*Δ (YSB3273), *hob1*Δ::*HOB1* (YSB4642), *sre1*Δ (YSB3315), *sre1*Δ::*SRE1* (YSB4727)]. Each strain was cultured in YPD broth at 30°C overnight, serially 10-fold diluted (1 to 10^4), and spotted on YPD or YP agar containing the indicated concentration of the following chemical agents (**a-f**): 1-1.5 M NaCl or KCl, 2M sorbitol, 30 mM hydroxyurea, 0.03% methy methanesulfonate (MMS), 2.0 mM diamide, 3.5 mM hydrogen peroxide (H_2O_2), 0.02 mM menadione, 15 mM dithiothreitol (DTT), 5 mg ml^{-1} calcofluor white (CFW), 1% Congo red, 0.015% sodium dodecyl sulphate (SDS), 2 µg ml^{-1} fludioxonil, 600 µg ml^{-1} 5-flucytosine, amphotericin B (0.6 and 0.8 µg ml^{-1}), and fluconazole (5 and 15 µg ml^{-1}). Each plate was incubated at 30°C and photographed after 2-5 days. To determine thermotolerance, cells were spotted on YPD medium and further incubated at 25°C, 30°C, 37°C, 39°C for 2 days and photographed. These spot assays were repeated more than three times, and one representative image was shown here. (**g**) Melanin assay with *hob1*Δ and *sre1*Δ mutants (*hob1* and *sre1*) and complemented strains (*HOB1C* and *SRE1C*)

in the pathogenic *Cryptococcus* complex. Each strain was spotted on Niger seed medium containing glucose, incubated at 30°C, and photographed for 4 days. This melanin assay was repeated more than three times, and one representative image was shown here. (**h**) Capsule production with *hob1*Δ and *sre1*Δ mutants in the pathogenic *Cryptococcus* complex. The relative packed cell volume was measured by calculating the ratio of the length of packed cell volume phase to the length of the total volume phase. Three independent experiments were performed (n=3). Data are presented as mean values ± SEM.

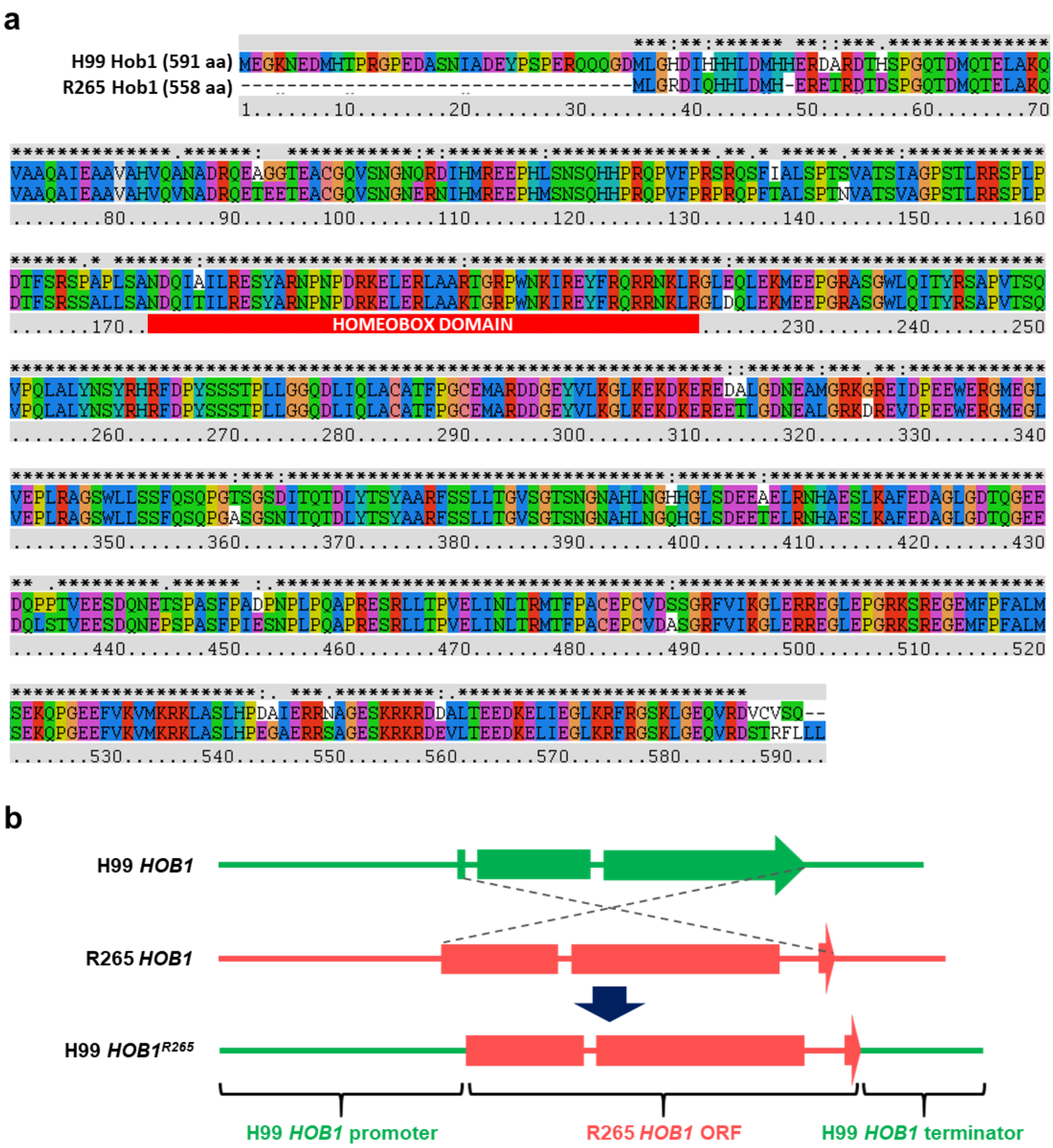

Supplementary Figure 9. Hob1 in *C. neoformans* and *C. deuterogattii*. (**a**) Amino acid alignment by using Cluster X. The sequence and domain information were obtained from UniProtKB (https://www.uniprot.org/) (**b**) Schematic diagram for *HOB1* gene swapping between *C. neoformans* H99 and *C. deuterogattii* R265 strains.

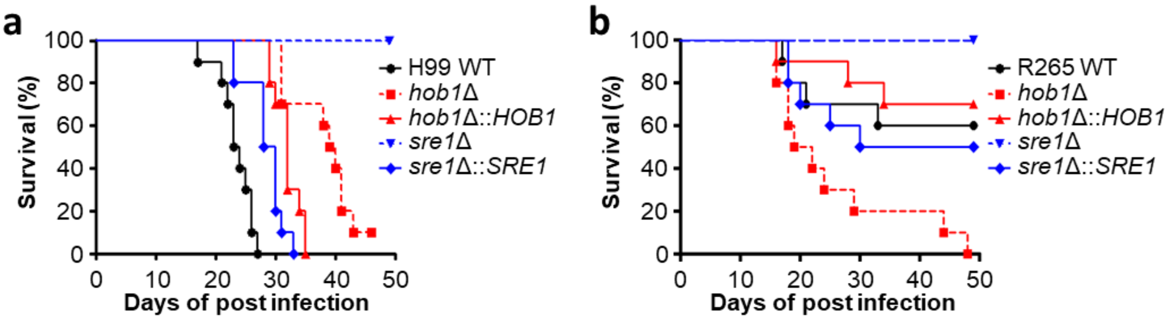

P value	WT	*hob1Δ::HOB1*	*sre1Δ::SRE1*
WT	-	<0.0001	0.361
hob1Δ	<0.0001	0.0048	-
sre1Δ	<0.0001	-	<0.0001

P value	WT	*hob1Δ::HOB1*	*sre1Δ::SRE1*
WT	-	0.611	0.694
hob1Δ	0.007	0.0009	-
sre1Δ	0.029	-	0.011

Supplementary Figure 10. Roles of Hob1 and Sre1 in virulence of *C. neoformans* and *C. deuterogattii*. C57BL/6 mice were infected with (**a**) *C. neoformans* WT (H99), *hob1*Δ, *hob1*Δ::*HOB1*, *sre1*Δ, and *sre1*Δ::*SRE1* strains constructed in H99 strain background, or (**b**) *C. deuterogattii* WT (R265), *hob1*Δ, *hob1*Δ::*HOB1*, *sre1*Δ, and *sre1*Δ::*SRE1* strains constructed in R265 background by intranasal instillation. Survival was monitored for 48 dpi and statistical significance was determined using the Mantel-Cox test by comparing a pair of indicated groups (ten mice per group, n=10).

Supplementary References

1 Lee, K. T. *et al.* Systematic functional analysis of kinases in the fungal pathogen *Cryptococcus neoformans*. *Nat Commun* **7**, 12766 (2016).

2 Jung, K. W. *et al.* Systematic functional profiling of transcription factor networks in *Cryptococcus neoformans*. *Nat Comms* **6**, 6757 (2015).
