# Supplementary material for: Fungal kinases and transcription factors regulating brain infection in Cryptococcus neoformans
Source: Nat Commun. 2020 Mar 23;11:1521. doi: 10.1038/s41467-020-15329-2 (PMC7090016; doi:10.1038/s41467-020-15329-2)
Supplement: Supplementary file 3 — Description of Additional Supplementary Files [file 41467_2020_15329_MOESM3_ESM.pdf]

Description of Additional Supplementary Files

File Name: Supplementary Data 1
Description: **In vivo gene expression profiling of *Cryptococcus neoformans* kinases and transcription factors via NanoString-nCounter platform.** NanoString™-nCounter®-based in vivo transcription analysis of 180 transcription factors, 183 kinases, and 58 known virulence-related genes, in *C. neoformans*. This assay was performed using total RNA extracted from the infected tissues (lungs, brain, spleen, and kidneys) recovered after 3, 7, 14, and 21 days post infection (dpi) from a murine cryptococcosis model by intranasal instillation and gene-specific nCounter codesets. Each in vivo gene expression level was normalised by average expression levels of 8 housekeeping genes (CNAG_00279, CNAG_00816, CNAG_02722, CNAG_02991, CNAG_00483, CNAG_01840, CNAG_03787, CNAG_04828) in the same tissue sample. Three A/J mice per dpi cohort were used (total 12 mice).

File Name: Supplementary Data 2
Description: **Brain-infection related kinases and TFs in *Cryptococcus neoformans.*** Kinases and TFs were summarised and classified by using functional description in database resources of *C. neoformans* (FungiDB, https://fungidb.org/fungidb/) and *S. cerevisiae* (SGD, https://www.yeastgenome.org/). ND indicates not-determined due to the growth defects in deletion mutants at human body temperature. IV indicates intravenous injection. ICV indicates intracerebroventricular injection. Asterisk indicates $P<0.05$ of STM study in Supplementary Figure 1-2 and Figure 4, and BBB transmigration assay in Figure 3.

File Name: Supplementary Data 3
Description: **Core-virulence regulating kinases and TFs in *Cryptococcus neoformans*.** Kinases and TFs were summarised and classified by using functional description in database resources of *C. neoformans* (FungiDB, https://fungidb.org/fungidb/) and *S. cerevisiae* (SGD, https://www.yeastgenome.org/). ND indicates not-determined due to the growth defects in deletion mutants at human body temperature. IV indicates intravenous injection. ICV indicates intracerebroventricular injection. Asterisk indicates $P<0.05$ of STM study in Supplementary Figure 1-2 and Figure 4, and BBB transmigration assay in Figure 3.

File Name: Supplementary Data 4
Description: **Strains used in this study.** Each *NAT-STM*#number indicates the NAT^r marker with a unique signature tag. ICV-STM of brain-virulence group was performed with each set of kinases deletion mutants (YSB1709, YSB3056, YSB3153, YSB2072, YSB1571, YSB3824, YSB1234, YSB2826 for STM-set 1; YSB1807, YSB3172, YSB1410, YSB123, YSB342, YSB3229, YSB1940 for STM-set 2;YSB4269, YSB3051, YSB4327, YSB330 for STM-set 3) and TF deletion mutants (YSB1980, YSB1585, YSB676, YSB1263, YSB1249, YSB1592 for STM-set 1; YSB2702, YSB2329, YSB693, YSB2447, YSB1820, YSB2381 for STM-set 2; YSB1104, YSB1013, YSB2108, YSB2089, YSB813 for STM-set 3) with *ste50*Δ mutant

(YSB3741) published previously (Supplementary reference 1 and 2). ICV-STM of core-virulence group was performed with kinases/TF deletion mutants (YSB2892, YSB3930, YSB273, YSB4275, YSB3632, YSB2216, YSB1800, YSB619, YSB64, YSB2157, YSB188, YSB2915, YSB2372, YSB2136, YSB2806, YSB2952, YSB3702, YSB2408, YSB552, YSB1883, YSB1904, YSB3814, YSB2415, YSB1968, YSB3093 for STM-set 1; YSB1266, YSB3714, YSB4341, YSB1564, YSB2040, YSB3063, YSB3329, YSB264, YSB4268, YSB3038, YSB1912, YSB510, YSB3096, YSB2308, YSB2387, YSB2984, YSB2493, YSB3300, YSB723 for STM-set 2) with *ste50*Δ mutant (YSB3741) published previously (Supplementary reference 1 and 2).

File Name: Supplementary Data 5
Description: **Primers used in this study.** Primer name and sequence (5’ to 3’ direction) were listed with each description.
