# Supplementary material for: Fungal kinases and transcription factors regulating brain infection in Cryptococcus neoformans
Source: Nat Commun. 2020 Mar 23;11:1521. doi: 10.1038/s41467-020-15329-2 (PMC7090016; doi:10.1038/s41467-020-15329-2)
Supplement: Supplementary file 9 — Reporting Summary [file 41467_2020_15329_MOESM9_ESM.pdf]

nature research

Corresponding author(s): Yong-Sun Bahn and Eunji Cheong

Last updated by author(s): Feb 27, 2020

Reporting Summary

Nature Research wishes to improve the reproducibility of the work that we publish. This form provides structure for consistency and transparency in reporting. For further information on Nature Research policies, see Authors & Referees and the Editorial Policy Checklist .

Statistics

For all statistical analyses, confirm that the following items are present in the figure legend, table legend, main text, or Methods section.

n/a	Confirmed	
☐	☒	The exact sample size (*n*) for each experimental group/condition, given as a discrete number and unit of measurement
☐	☒	A statement on whether measurements were taken from distinct samples or whether the same sample was measured repeatedly
☐	☒	The statistical test(s) used AND whether they are one- or two-sided<br>*Only common tests should be described solely by name; describe more complex techniques in the Methods section.*
☐	☒	A description of all covariates tested
☐	☒	A description of any assumptions or corrections, such as tests of normality and adjustment for multiple comparisons
☐	☒	A full description of the statistical parameters including central tendency (e.g. means) or other basic estimates (e.g. regression coefficient) AND variation (e.g. standard deviation) or associated estimates of uncertainty (e.g. confidence intervals)
☒	☐	For null hypothesis testing, the test statistic (e.g. *F*, *t*, *r*) with confidence intervals, effect sizes, degrees of freedom and *P* value noted<br>*Give P values as exact values whenever suitable.*
☒	☐	For Bayesian analysis, information on the choice of priors and Markov chain Monte Carlo settings
☒	☐	For hierarchical and complex designs, identification of the appropriate level for tests and full reporting of outcomes
☒	☐	Estimates of effect sizes (e.g. Cohen's *d*, Pearson's *r*), indicating how they were calculated

Our web collection on statistics for biologists contains articles on many of the points above.

Software and code

Policy information about availability of computer code

Data collection	None.
Data analysis	Statistical analyses were performed with GraphPad Prism version 8.<br>NanoString data was normalised by nSolver software (provided by NanoString).

For manuscripts utilizing custom algorithms or software that are central to the research but not yet described in published literature, software must be made available to editors/reviewers. We strongly encourage code deposition in a community repository (e.g. GitHub). See the Nature Research guidelines for submitting code & software for further information.

Data

Policy information about availability of data

All manuscripts must include a data availability statement. This statement should provide the following information, where applicable:

- Accession codes, unique identifiers, or web links for publicly available datasets
- A list of figures that have associated raw data
- A description of any restrictions on data availability

The whole STM-based murine data for all kinase and TF mutants are provided in Supplementary Figs. 1 and 2. For the NanoString nCounter® analysis, the whole in vivo kinase and TF gene expression data and probe information are included in Supplementary Data 1. All the sequence and protein domain information was obtained from FungiDB (https://fungiDB.org) and UniProtKB (https://www.uniprot.org/), respectively. The data that support the findings of this study are available from the corresponding authors upon reasonable request. The source data underlying Supplementary Figs 5, 6, and 7 are provided as a Source Data file.

Field-specific reporting

Please select the one below that is the best fit for your research. If you are not sure, read the appropriate sections before making your selection.

[x] Life sciences [ ] Behavioural & social sciences [ ] Ecological, evolutionary & environmental sciences

For a reference copy of the document with all sections, see nature.com/documents/nr-reporting-summary-flat.pdf

Life sciences study design

All studies must disclose on these points even when the disclosure is negative.

Sample size

Three mice were used in each group for STM and NanoString experiments. We chose to use three mice because it can result in a statistically significant outcome, as shown in previous studies (Jung et al. Nat Commun 2015 6:6757; Lee et al. Nat Commun 2016 7:12766). We did not use more than three mice for single group because of the cost and ethical reasons.

For the murine survival assay, seven (A/J) or ten (C57BL/6) mice per each group were used to compare the virulence of WT and mutant strains.

For the in vitro analysis including BBB crossing/adhesion assay and quantitative PCR, we used more than three-independent biological samples to perform statistics analysis.

Data exclusions

None.

Replication

For quantitative PCR and BBB crossing/adhesion assays, three biologically independent experiments with duplicates were performed. We indicated all data points in figures.

For diagnostic PCR, Southern blot analysis, spot assay, and virulence factor analysis, we performed more than two independent experiments to confirm the reproducibility. If data show similar patterns, we exhibited one representative image of two independent experiments.

Randomization

For the murine analysis, a mouse was randomly chosen for each cohort.

Blinding

The investigators were not blinded during data collection and analysis. But all experiments were performed without any discrimination.

Reporting for specific materials, systems and methods

We require information from authors about some types of materials, experimental systems and methods used in many studies. Here, indicate whether each material, system or method listed is relevant to your study. If you are not sure if a list item applies to your research, read the appropriate section before selecting a response.

Materials & experimental systems

n/a	Involved in the study	
[x]	[ ]	Antibodies
[ ]	[x]	Eukaryotic cell lines
[x]	[ ]	Palaeontology
[ ]	[x]	Animals and other organisms
[x]	[ ]	Human research participants
[x]	[ ]	Clinical data

Methods

n/a	Involved in the study	
[x]	[ ]	ChIP-seq
[x]	[ ]	Flow cytometry
[x]	[ ]	MRI-based neuroimaging

Eukaryotic cell lines

Policy information about cell lines

Cell line source(s)	Human brain microvascular endothelial cell (HBMEC) line (hCMEC/D3 cell line, Merck)
Authentication	None.
Mycoplasma contamination	Not tested for mycoplasma contamination
Commonly misidentified lines (See ICLAC register)	None.

Animals and other organisms

Policy information about studies involving animals; ARRIVE guidelines recommended for reporting animal research

Laboratory animals	Seven-week-old female A/J mice (Japan SLC, Inc.); Seven- to eight-week-old female C57BL/6 mice (BioLASCO Taiwan Co., Ltd)<br><br>The mice were maintained with free access to food and water under a 12-h light and 12-h dark cycle, with the light cycle beginning at 7:00 a.m.
Wild animals	None.
Field-collected samples	None.
Ethics oversight	Animal care and all experiments were conducted in accordance with the ethical guidelines of the Institutional Animal Care and Use Committee (IACUC) of Yonsei University and National Taiwan University. The Yonsei University and National Taiwan University IACUC approved all of the vertebrate studies.

Note that full information on the approval of the study protocol must also be provided in the manuscript.
